# Crystal structures of 40- and 71-substitution variants of hydroxynitrile lyase from rubber tree

**DOI:** 10.1107/S2059798325007065

**Published:** 2025-08-27

**Authors:** Colin T. Pierce, Panhavuth Tan, Lauren R. Greenberg, Meghan E. Walsh, Ke Shi, Alana H. Nguyen, Elyssa L. Meixner, Sharad Sarak, Hideki Aihara, Robert L. Evans, Romas J. Kazlauskas

**Affiliations:** a Department of Biochemistry, Molecular Biology and Biophysics University of Minnesota Minneapolis MN55455 USA; National Hellenic Research Foundation, Greece

**Keywords:** engineered proteins, hydroxynitrile lyases, α/β-hydrolase fold, esterases, SABP2, salicylic acid-binding protein 2

## Abstract

Hydroxynitrile lyase from *Hevea brasiliensis* (*Hb*HNL) and the esterase SABP2 from *Nicotiana tabacum* share the α/β-hydrolase fold, a Ser–His–Asp catalytic triad and 44% sequence identity, yet catalyze different reactions. Prior studies showed that three active-site substitutions in *Hb*HNL conferred weak esterase activity. To investigate how regions beyond the active site influence catalytic efficiency and active-site geometry, we engineered *Hb*HNL variants with increasing numbers of substitutions to match SABP2. Variant HNL16 has all amino acids within 6.5 Å of the active site identical to SABP2, HNL40 those within 10 Å and HNL71 those within 14 Å. HNL16 exhibited poor esterase activity, whereas both HNL40 and HNL71 showed efficient esterase catalysis, demonstrating that residues beyond the immediate active site are critical for functional conversion. X-ray structures of HNL40 and HNL71 reveal a progressive shift in backbone positions toward those of SABP2, with r.m.s.d. values of 0.51 Å (HNL40) and 0.41 Å (HNL71) over the C^α^ atoms, and even smaller r.m.s.d.s within the active-site region. Both HNL40 and HNL71 show a restored oxyanion hole and an additional tunnel connecting the active site to the protein surface. This work demonstrates the essential role of distant, indirectly acting residues to catalysis in α/β-hydrolase enzymes.

## Abstract 2

Variants HNL16, HNL40 and HNL71 of hydroxynitrile lyase from *Hevea brasiliensis* contain 16, 40 and 71 mutations, respectively, to make increasingly larger regions surrounding the active site identical in sequence to the esterase SABP2. X-ray structures of HNL40 and HNL71 reveal changes to the oxyanion hole and a new tunnel, which may contribute to their efficient esterase activity.

## 1. Introduction

**Figure 1:**
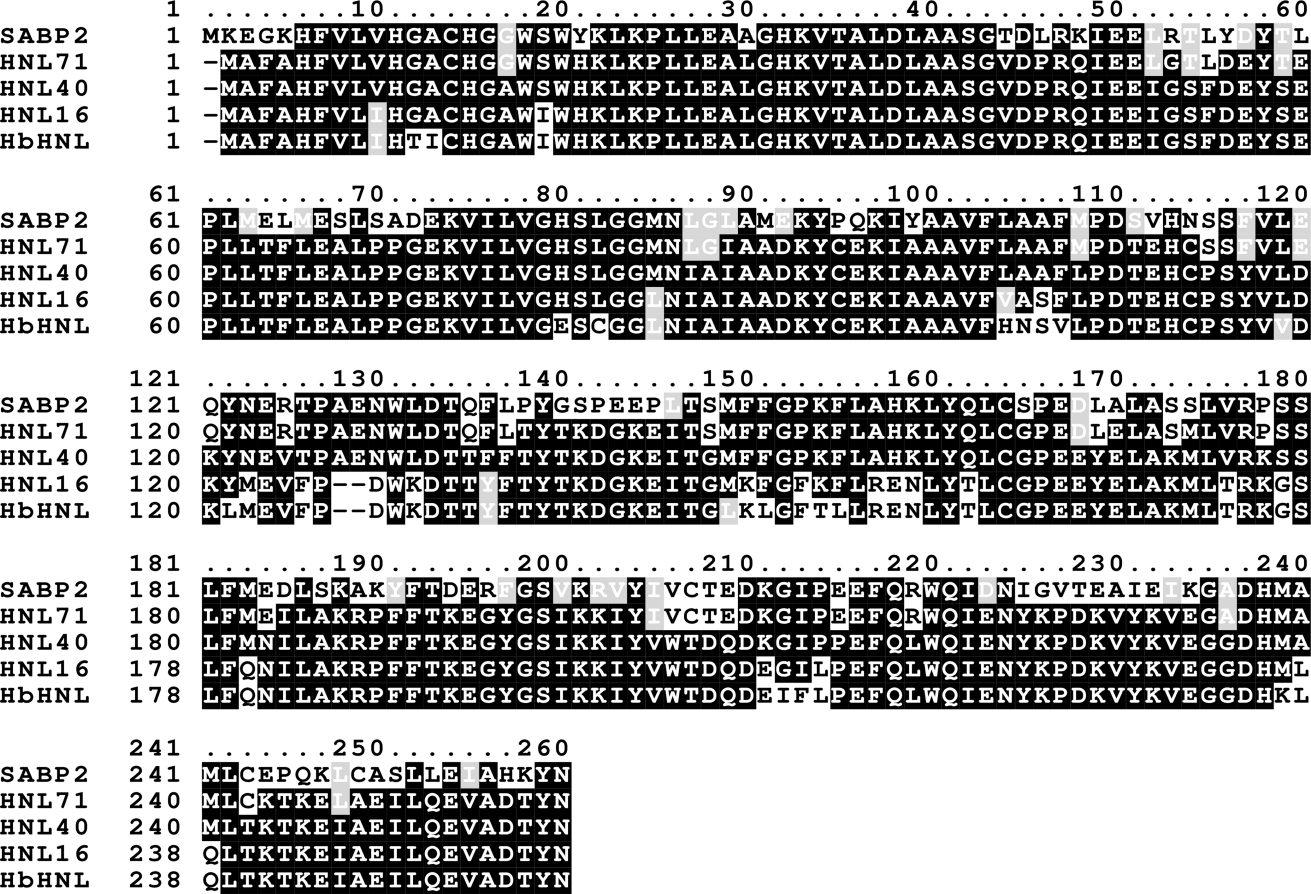
Sequence alignment of HNL16, HNL40 and HNL71 with the starting sequence *Hb*HNL and the target sequence SABP2. Positions with identical amino acids are shaded black, positions with similar amino acids are shaded gray and positions with dissimilar amino acids are unshaded. The catalytic triad is conserved in all four proteins and occurs at Ser80–His237–Asp209 in the HNL40/71 numbering. Sequences were aligned with *Clustal Omega* (Sievers *et al.*, 2011 ▸) and shaded with *Boxshade* (https://junli.netlify.app/apps/boxshade/). HNL16, HNL40 and HNL71 also contained an N- or C-terminal His_6_-tag which is not shown in this alignment

Hydroxynitrile lyase from the rubber tree *Hevea brasiliensis* (*Hb*HNL) and SABP2, an esterase from tobacco, are homologs with 44% sequence identity (114 identical residues, 146 differing over 260 positions; Fig. 1 ▸). The two proteins have the same α/β-hydrolase protein fold and the same Ser–His–Asp catalytic triad.

**Figure 2:**
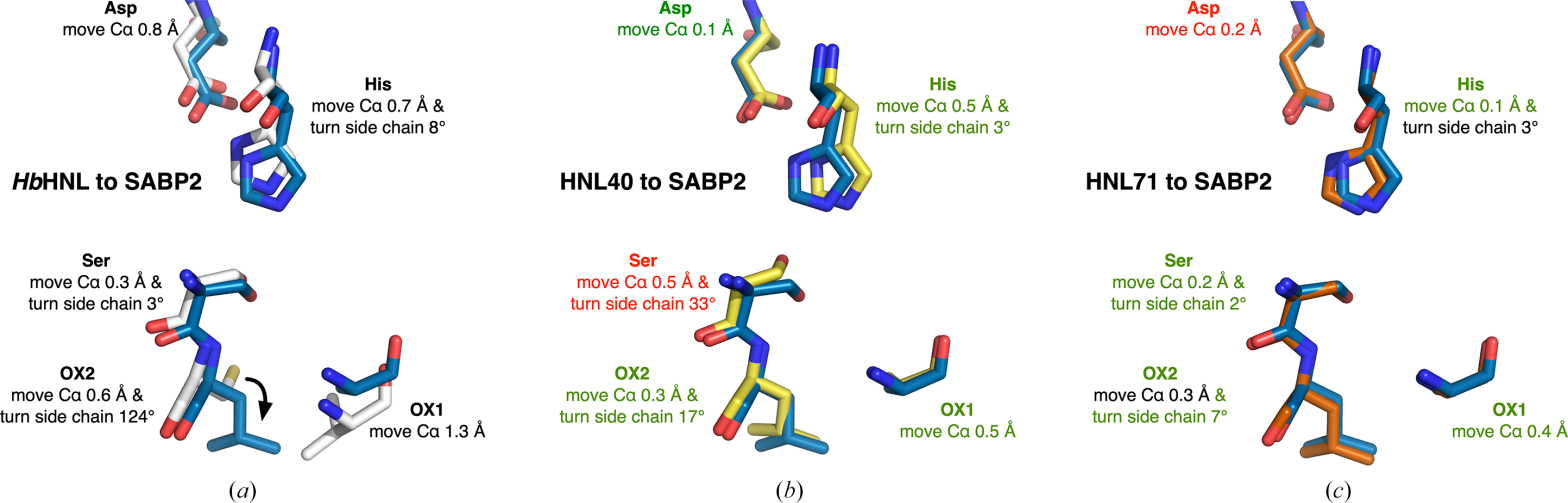
Overlay of the catalytic residues of (*a*) *Hb*HNL (PDB entry 3c6x), (*b*) HNL40 (PDB entry 8sni) and (*c*) HNL71 (PDB entry 9clr) onto the esterase SABP2 (PDB entry 1y7h, blue C atoms). The alignment of structures minimized the r.m.s.d. between 205–224 of the 256 corresponding C^α^-atom pairs using the *align* function in *PyMOL*. The text indicates the structural changes needed for the HNL residues to match the structures of the SABP2 residues. Green text marks changes the improve the match, while red text marks changes that increase the differences. (*a*) The C^α^ positions in *Hb*HNL differ by 0.3–1.3 Å from those in SABP2. The OX1 residue shows the largest deviation. The side-chain conformations are similar, except for OX2, which differs by 124° in the χ_1_ angle. (*b*) In HNL40, four of the five residues have moved closer to their positions in SABP2, but the serine C^α^ position and χ_1_ angle have moved further away. (*c*) In HNL71 the C^α^ positions differ by only 0.1–0.4 Å and the χ_1_ angles differ by less than 7° from those in SABP2. The displacement of the C^α^ position of the aspartate increases from 0.1 Å in HNL40 to 0.2 Å in HNL71.

Despite these similarities, a comparison of the catalytic residues in *Hb*HNL and SABP2 reveals an apparently non­functional oxyanion hole in *Hb*HNL (Fig. 2 ▸*a*). The overall C^α^-atom positions in *Hb*HNL differ from those in SABP2 by an average of 0.69 Å for the best 218 comparisons out of a total of 256 atom pairs. The C^α^-atom positions of the catalytic triad (Ser81, His238 and Asp210; SABP2 numbering) and oxyanion hole (Ala13 and Leu82) differ by 0.3–1.3 Å, with the largest difference at OX1. This large difference in C^α^ position at OX1 is accompanied by a similar difference between the catalytic N-atom positions in *Hb*HNL and SABP2. A second problem with the oxyanion hole in *Hb*HNL is the side-chain orientation of OX2. The Cys81 side chain in *Hb*HNL points into the oxyanion-binding region and would prevent binding. In SABP2 the χ_1_ angle for Leu82 differs by 124° and places the side chain outside the binding site. The combination of a hindering side chain at OX2 and a main-chain position differing by 1.3 Å at OX1 is expected to render the oxyanion hole nonfunctional in *Hb*HNL. A nonfunctional oxyanion hole in *Hb*HNL is consistent with its proposed mechanism, which requires binding of the cyanohydrin outside the oxy­anion hole region. A functional oxyanion hole in *Hb*HNL would be expected to slow catalysis due to nonproductive binding of the substrate. We hypothesized that adding substitutions (and insertions) to *Hb*HNL from SABP2 in the regions closest to the active-site region would make the catalytic residue positions and orientations more similar to those in SABP2, thereby restoring the oxyanion hole and efficient esterase activity.

HNL16, HNL40 and HNL71 are variants of *Hb*HNL with 16, 40 and 71 changes, respectively, to make the protein sequence in the region surrounding the active site identical to that in the esterase SABP2. The active site of SABP2 is defined as the region surrounding a bound product molecule, salicylic acid, in the X-ray structure of SABP2 (PDB entry 1y7i).

24 amino-acid residues in SABP2 lie within 6.5 Å of the bound product salicylic acid in the X-ray structure (PDB entry 1y7i). Ten of these residues are identical in *Hb*HNL, so 14 amino-acid substitutions in *Hb*HNL would make all residues within 6.5 Å of the substrate-binding region identical to those in SABP2. Hydrogen bonds and van der Waals contacts occur within ∼4 Å, so the more permissive distance of 6.5 Å includes transient interactions that may occur as the protein moves. The substitutions include both oxyanion-hole residues: Ile12Ala and Cys81Leu. Two more distant substitutions, H103V (7.9 Å from salicylic acid) and T151K (11.1 Å from salicylic acid) were included to stabilize the protein. SABP2 contains leucine at the position corresponding to residue 103, so the replacement with valine is with a similar but not identical amino acid. HNL16 contains the following 16 substitutions: T11G, I12A, E79H, C81L, H103V, N104A, V106F, V118L, L121Y, L146M, L148F, T151K, L152F, I209G, F210I and K236M. Since there are 146 differences between the sequences of *Hb*HNL and SABP2, these 16 changes eliminate approximately 10% of the differences, increasing the overall sequence identity with SABP2 from 44% for *Hb*HNL to 50% for HNL16.

Variant HNL40 introduces 40 changes to *Hb*HNL to increase its sequence identity with SABP2 to 59%. The active-site region of SABP2 contains 59 residues with at least one atom within 10 Å of the bound salicylic acid. Of these, 27 residues are identical in SABP2 and *Hb*HNL, while 32 differ. To fully match the sequence of the active-site region, HNL40 incorporates substitutions for all 32 differing residues. HNL40 contains all of the changes in HNL16, except for H103V. Variants HNL40 and HNL71 have a leucine at this position so it matches the amino acid in SABP2. The 32 substitutions are as follows (*Hb*HNL numbering): I9V, T11G, I12A, I18S, E79H, C81L, L84M, H103L, N104A, S105A, V106F, V118L, L121Y, M122N, F125T, D127N, Y133F, L146M, K147F, L148F, L152F, N156K, T173V, G176S, Q180M, E208K, I209G, F210I, L211P, K236M, L237A and Q238M. HNL40 contains eight additional changes in the lid domain beyond this 10 Å region. There is an insertion of two residues, AE, after residue 126 and six additional substitutions (*Hb*HNL numbering): K129L, F150P, T151K, R154A, E155H and T159Q. Thus, HNL40 contains a total of 40 amino-acid changes relative to *Hb*HNL: 38 amino-acid substitutions and two inserted amino acids. In the HNL40 and HNL71 numbering, the numbering of the matching residues at positions 127 and above is two units higher than in *Hb*HNL due to the two amino-acid residue insertion after position 126.

Variant HNL71 contains 31 additional substitutions beyond those in HNL40, giving a total of 71, which corresponds to removing approximately one half (49%) of the differences between the proteins. These 31 additional substitutions increase the sequence identity to SABP2 to 71% for HNL71 and expand the region of identical protein sequence with SABP2 to 14 Å from salicylic acid bound in the active site of SABP2. There are 130 residues in SABP2 with at least one atom within 14 Å of the salicylic acid bound to the active site. 59 of these are within 10 Å, so there are 71 more residues to consider in the region from 10 to 14 Å. 32 of these 71 residues are identical between SABP2 and *Hb*HNL and 39 residues differ. Eight of these changes in the lid region were already included in HNL40 above, so only 31 additional substitutions are required to make the sequence identical to that in SABP2 within 14 Å of the active site. Those additional 31 substitutions are (*Hb*HNL numbering): A16G, I51L, S53T, F54L, S58T, I86L, A87G, L107M, P114S, Y116F, D119E, K120Q, V124R, T132Q, F134L, G145S, E165D, Y166L, K170S, K175P, N181E, V202I, W203V, T204C, D205T, Q206E, P212E, L216R, G233A, T240C and I245L.

The X-ray structures of HNL40 and HNL71 reveal the degree to which these substitutions increased the similarity of the catalytic atom positions to those in SABP2. The 40 changes in HNL40 make these positions similar, but not identical; the additional 31 substitutions in HNL71 make the catalytic atom positions identical to those in SABP2. The tunnels in HNL40 and HNL71 connect the active site, which contains residues identical to those in SABP2, and the enzyme surface, which contains residues differing from those in SABP2. The pattern of tunnels in HNL40 and HNL71 is intermediate between those in *Hb*HNL and SABP2.

## 2. Materials and methods

### 2.1. Protein production and purification

The HNL16, HNL40 and HNL71 genes containing a His_6_-tag either at the N-terminus (HNL40) or the C-terminus (HNL16 and HNL71) were codon-optimized, synthesized and cloned into pET-21a(+) vectors by Twist Biosciences (Table 1 ▸ and Supplementary Table S1). The gene for HNL16 encodes 268 amino acids (257 amino acids plus 11 amino acids to encode a C-terminal linker and His_6_-tag), that for HNL40 encodes 293 amino acids (259 amino acids plus 33 amino acids to encode an N-terminal linker and His_6_-tag) and the gene for HNL71 encodes 270 amino acids (259 amino acids plus 11 amino acids to encode a C-terminal linker and His_6_-tag). The longer linker for HNL40 also encodes, besides a His_6_-tag, a thrombin cleavage site and a T7 tag. Our experiments only used the His_6_-tag.

Lysogeny broth (LB) medium (5 ml) containing carbenicillin (100 µg ml^−1^) was inoculated with a single bacterial colony picked from an agar plate and incubated in an orbital shaker at 37°C and 240 rev min^−1^ for 15 h to create a seed culture. A 1 l baffled flask containing Terrific broth–carbenicillin medium (250 ml) was inoculated with 2.5 ml seed culture. This pre-induction culture was incubated at 37°C and 240 rev min^−1^ for 3–4 h until the absorbance at 600 nm reached 0.4–1.0. The flask was then cooled on ice for 30 min. Isopropyl β-d-1-thiogalactopyranoside (0.75–1.0 m*M* final concentration) was added to induce protein expression, and cultivation was continued for 20–24 h at 16°C. The cells were harvested by centrifugation (7000*g*, 15 min at 4°C), resuspended in Ni–NTA loading buffer (10 m*M* imidazole, 50 m*M* Tris pH 8.0, 500 m*M* NaCl; 4 ml per gram of wet cells) and either directly sonicated or frozen for storage and later purification. Cells were flash-frozen in liquid nitrogen or a dry ice–ethanol bath and stored at −80°C. Frozen cells were thawed at room temperature or in a room-temperature water bath, and fresh/thawed cells were disrupted by sonication (400 W, 40% amplitude for 3 min). The cell lysate was centrifuged to pellet the cell debris (4°C, 20 000*g* for 20 min), which was discarded.

The crude lysate was purified via nickel-affinity chromatography. The supernatant from above was mixed with 1–2.5 ml Ni–NTA resin (pre-equilibrated with 10 ml Ni–NTA loading buffer) and incubated for 45 min at 4°C with rotation (10 rev min^−1^). The resin/supernatant mixture was loaded onto a 25 ml gravity-flow column (Bio-Rad) and the resin was washed with ten column volumes each of buffers *A* and *B* for a total of 20 column volumes (50 m*M* Tris pH 8.0, 500 m*M* NaCl; buffer *A* contained 25 m*M* imidazole and buffer *B* contained 50 m*M* imidazole). The His-tagged protein was eluted with ten column volumes of elution buffer (125 m*M* imidazole, 50 m*M* Tris pH 8.0, 500 m*M* NaCl) and collected in 1 ml fractions. The protein concentration of each elution fraction was determined from its absorbance at 280 nm as measured using a NanoDrop 2000 (Thermo Scientific) and the calculated extinction coefficient for the protein (https://web.expasy.org/protparam/). Protein gels were used to check the presence and purity of the protein and were run using sodium dodecyl sulfate–polyacrylamide gradient gels (NuPage 4–12% Bis-Tris gel, Invitrogen) using the Precision Plus Dual Color protein standard (Bio-Rad, 5 µl per lane) for 50–60 min at 120 V, stained with SimplyBlue Safe Stain (Thermo Fisher Scientific) and destained twice with Milli-Q UltraPure water. SDS–PAGE indicated a molecular weight of ∼31 kDa, in agreement with the predicted weights of 32.8 kDa for the HNL40 construct and 30.4 kDa for the HNL71 construct (Supplementary Fig. S1). Only the most concentrated elution fractions, typically fractions 2–10, were pooled to reduce the presence of contaminating proteins and then sterile-filtered. The imidazole-containing elution buffer was exchanged by the addition of BES buffer [5 m*MN*,*N*-bis(2-hydroxyethyl)-2-aminoethanesulfonic acid (BES) pH 7.2, 14 ml] followed by ultrafiltration (Amicon 15 ml ultrafiltration centrifuge filter, 10 kDa cutoff) to reduce the volume to ∼1 ml. This addition of buffer and filtration was repeated four times. The last ultrafiltration spin was extended by 10 min to reduce the volume to ∼250 µl. The final protein concentration was determined via spectrophotometric measurements at 280 nm, measured in duplicate and averaged. A 250 ml culture typically yielded 2–5 mg protein.

Samples used for crystallization were further purified via size-exclusion chromatography. The purification column (Cytiva HiLoad 16/600 Superdex 200 pg, 120 ml capacity) was equilibrated with 1.10 column volumes of running buffer (5 m*M* BES pH 7.2) prior to sample injection. The sample was injected and eluted at a flow rate of 1 ml min^−1^ for 1.10 column volumes of running buffer. 3 ml fractions were collected in 15 ml conical tubes when the *A*_280_ signal intensity exceeded 15 mAU (absorbance units). HNL71 eluted at 65–75 min and the signal intensity peaked at ∼70 min, while that for HNL40 peaked at ∼64 min (Supplementary Fig. S2). Elution fractions 2–6 (HNL71) and 1–5 (HNL40) were evaluated for purity, sterile-filtered, pooled and concentrated as above. The final, purified protein concentrations were 12 mg ml^−1^ (HNL71) and 8 mg ml^−1^ (HNL40).

### 2.2. Steady-state kinetics of the catalysis of ester hydrolysis

Ester hydrolysis was measured at 404 nm using *p*-nitrophenyl acetate (*p*NPAc), which releases the yellow *p*-nitrophenoxide. The reaction mixture (100 µl; path length 0.29 cm) contained 0.03–2.4 m*Mp*NPAc, 7–8%(*v*/*v*) acetonitrile, 5 m*M* BES buffer pH 7.2 and up to 15 µg enzyme (5 µ*M*). The slope of increase in absorbance versus time was measured in triplicate, fitted to a line using linear regression and corrected for spontaneous hydrolysis of *p*NPAc with blank reactions lacking protein. The extinction coefficient used for calculations (ɛ_404 nm_ = 16 600 cm^–1^ *M*^–1^) accounts for the incomplete ionization of *p*-nitrophenol at pH 7.2. The enzyme concentration was determined by the average absorbance at 280 nm measured in duplicate and normalized by subtracting a buffer blank. The *k*_cat_ and *K*_m_ were determined using a nonlinear fit of the experimental data to the Michaelis–Menten equation using the *Solver* program in *Microsoft Excel* or using the statistical program *R* (R Core Team; https://www.r-project.org/) with an enzyme-kinetics script (Huitema & Horsman, 2018 ▸).

### 2.3. Crystallization

Crystallographic screening was performed using the Crystal Phoenix Dispenser (Art Robbins Instruments/Hudson Robotics). Sitting-drop trays and CrystalMation Intelli-Plate low-profile 96-well plates from Hampton Research were used for the crystallization setup. All of the setup and washing procedures were performed using the Phoenix software. The proteins were crystallized within a few days of purification and the data were collected within 2–4 weeks.

For HNL40, each crystallization drop consisted of 0.1 µl protein sample (8 mg ml^−1^ protein) mixed with 0.1 µl well solution (Table 2 ▸). A total of 960 conditions were screened. Crystals formed within one day from the Index HT screen from Hampton Research under the condition 0.1 *M* 4-(2-hydroxyethyl)-1-piperazineethanesulfonic acid (HEPES) pH 7.5, 0.2 *M*l-proline, 10%(*w*/*v*) polyethylene glycol 3350 and grew to a full size of 160 × 50 × 10 µm in three days. The cryoprotectant solution for the HNL40 crystals comprised 0.1 *M* HEPES pH 7.5, 0.2 *M*l-proline, 10%(*w*/*v*) polyethylene glycol 3350, 25%(*v*/*v*) ethylene glycol. This robotic screening produced crystals that were sufficient for data collection.

For HNL71, each crystallization drop consisted of 0.1 µl protein sample (12 mg ml^−1^ protein) mixed with 0.1 µl well solution (Table 2 ▸). A total of 960 conditions were screened. Crystals formed within one day from the Index HT screen from Hampton Research under the condition 0.1 *M* Tris pH 8.5, 0.2 *M* magnesium chloride hexahydrate, 25%(*w*/*v*) polyethylene glycol 3350 and grew to a full size of 50 × 80 × 30 µm in three days. The cryoprotectant solution for the HNL71 crystals comprised 0.1 *M* Tris pH 8.5, 0.2 *M* magnesium chloride hexahydrate, 25%(*w*/*v*) polyethylene glycol 3350, 25%(*v*/*v*) ethylene glycol. This robotic screening produced crystals sufficient for data collection.

### 2.4. Data collection, processing and structure refinement

The X-ray diffraction data for HNL40 were collected on beamline 24-ID-C at the Advanced Photon Source (APS), Lemont, Illinois, USA using a wavelength of 0.979 Å (Table 3 ▸). Crystals were preserved by flash-cooling in liquid nitrogen with a cryoprotectant solution comprised of 0.1 *M* HEPES pH 7.5, 0.2 *M*l-proline, 10%(*w*/*v*) polyethylene glycol 3350, 25%(*v*/*v*) ethylene glycol. Data were collected at 100 K with a Dectris EIGER2 S 16M detector, an exposure time of 0.2 s per frame, a crystal-to-detector distance of 180 mm and an oscillation angle of 0.2° per frame. The crystal belonged to space group *P*2_1_2_1_2_1_ with unit-cell dimensions *a* = 76.53, *b* = 81.88, *c* = 90.93 Å. The data set was 98.04% complete to a resolution of 1.99 Å, with an overall *R*_merge_ of 0.066 and a multiplicity of 3.70. The Matthews coefficient was 2.17 Å^3^ Da^−1^ and the solvent content was 43.37%.

The X-ray diffraction data for HNL71 were collected on beamline BL12-2 at the Stanford Synchrotron Radiation Lightsource (SSRL) using a wavelength of 0.9793 Å. The crystals were preserved by flash-cooling in liquid nitrogen using a cryoprotectant solution composed of 0.1 *M* Tris pH 8.5, 0.2 *M* magnesium chloride hexahydrate, 25%(*w*/*v*) polyethylene glycol 3350, 25%(*v*/*v*) ethylene glycol. Data were collected at 100 K with an EIGER 16M PAD detector, an exposure time of 0.5 s per frame, a crystal-to-detector distance of 270 mm and an oscillation angle of 0.5° per frame. The crystal that generated the data set belonged to space group *P*2_1_2_1_2_1_ with unit-cell dimensions *a* = 49.97, *b* = 80.75, *c* = 135.46 Å. The data set was 94.64% complete to a resolution of 1.99 Å, with an overall *R*_merge_ of 0.095 and a multiplicity of 5.98. The Matthews coefficient was 2.24 Å^3^ Da^−1^ and the solvent content was 45.14%. The final data-collection and processing statistics are summarized in Table 3 ▸.

The data for both crystals were processed using *HKL*-2000 (Otwinowski & Minor, 1997 ▸). The initial structure of HNL40 was obtained via molecular replacement using the structure of hydroxynitrile lyase from rubber tree with seven mutations (PDB entry 8euo; Pierce *et al.*, 2025 ▸) as the search model with an inputted sequence identity of 84.5%. The initial structure of HNL71 was obtained via molecular replacement using the predicted structure of HNL71 from *AlphaFold*2 (Jumper *et al.*, 2021 ▸), which was used as the search model with an inputted sequence identity of 100%. This 100% value ignores the C-terminal His_6_-tag in HNL71, which was not resolved in the electron density. Molecular replacement was performed with *Phaser* (McCoy *et al.*, 2007 ▸) in the *Phenix* software suite version 1.19.2 (Liebschner *et al.*, 2019 ▸). Subsequent rounds of refinement were carried out using *phenix.refine* and manual adjustment of the initial model using *Coot* (Emsley *et al.*, 2010 ▸). The *PDB-REDO* software (Joosten *et al.*, 2014 ▸) was used at an intermediate refinement stage (round 66) for HNL71 and was followed by additional manual refinement to ensure a chemically reasonable structure. *PDB-REDO* was not used during the refinement of the HNL40 structure.

Tables 3 ▸ and 4 ▸ show the detailed data-collection, processing and refinement statistics for both proteins. The figures of the protein structure in this paper were created using *PyMOL* v.2.5.4 or v.3.03 (Schrödinger; https://pymolwiki.org). The final structure models were deposited in the Research Collaboratory for Structural Bioinformatics Protein Data Bank as PDB entry 8sni for HNL40 and PDB entry 9clr for HNL71.

The three SABP2 structures (PDB entries 1xkl, 1y7h and 1y7i) used for catalytic atom position comparison in Table 5 ▸ were crystallized under different conditions. These structures have different space groups, contain different active-site ligands and were refined to resolutions ranging from 2.00 to 2.52 Å. In contrast, the three *Hb*HNL structures (PDB entries 6yas, 3c6x and 2g4l) were crystallized under identical conditions, yielding crystals in the same space group. All three are apo structures (without bound ligands). However, they differ in resolution and data-collection temperature: PDB entry 6yas has lower resolution (2.20 Å) with room-temperature data collection, while the other two structures (PDB entries 2g4l and 3c6x) have higher resolutions (1.84 and 1.05 Å, respectively) with data collected at low temperature (100 K). PDB entry 6yas was included in the comparison to better match the variability in crystallization conditions observed in the SABP2 structure set.

## 3. Results and discussion

### 3.1. Esterase activity of variants

Wild-type *Hb*HNL shows a low promiscuous esterase activity towards *p*-nitrophenyl acetate, with a *k*_cat_ value of 0.25 min^−1^, which is 520-fold lower than that for the esterase SABP2 (130 min^−1^; Table 5 ▸). Previous work identified HNL3, which contains three substitutions in the active site (T11G, E79H and K236G) and shows more than a sixfold higher *k*_cat_ (1.6 min^−1^). Increasing the number of substitutions to 16 in HNL16 surprisingly decreased the catalytic turnover to 0.35 min^−1^. HNL16 contains two of the three substitutions in HNL3: T11G and E79H. The third substitution, K236G, is replaced with K236M to match the sequence of SABP2. Some esterases contain a glycine at this position.

Both HNL40 and HNL71 show dramatically increased esterase activity. The *k*_cat_ value for HNL40 (74 min^−1^) is 300-fold higher than that for *Hb*HNL and only approximately twofold lower than SABP2. The 31 additional substitutions in HNL71 further increase the *k*_cat_ value fourfold to 310 min^−1^, making HNL71 a 2.4-fold better esterase than SABP2. The *K*_m_ values range from 0.6 to 7 m*M*, with the exception of HNL3, which has a lower *K*_m_ value of 0.16 m*M*. Although the sequence of HNL71 is closer to that of *Hb*HNL (72% sequence identity) by ten amino acids than to SABP2 (68% sequence identity), it shows no significant hydroxynitrile lyase activity and instead is a 2.4-fold better esterase than SABP2.

### 3.2. Crystallization and structure determination

To identify possible structural reasons for the increased esterase activity of HNL40 and HNL71, we solved their X-ray crystal structures. Molecular replacement using the X-ray structure of a homolog (84.5% sequence identity, PDB entry 8euo) generated an initial structure of HNL40, and refinement based on 39 116 reflections, with 2007 reflections (5%) set aside for *R*_free_, yielded a final structure with an *R*_work_ of 0.1991 and an *R*_free_ of 0.2233. The asymmetric unit contained two protein monomers, as did the that of the target protein SABP2 (PDB entry 1y7i; Forouhar *et al.*, 2005 ▸). The asymmetric unit of the parent protein *Hb*HNL contains a single chain (PDB entry 1yb6; Wagner *et al.*, 1996 ▸), but it forms a dimer in solution, most likely corresponding to a crystallographic dimer. The HNL40 dimer consists of 517 protein residues, 68 solvent molecules and a total of 4013 non-H atoms. The average *B* factor for the final model was 38.61 Å^2^. Structural validation using *MolProbity* showed 97.27% of the residues in the favored regions and 2.73% in the allowed regions of the Ramachandran plot, with no outliers (Table 4 ▸).

Molecular replacement using an *AlphaFold*2 (Jumper *et al.*, 2021 ▸) model of HNL71 generated an initial structure of HNL71 with an LLG of 3623.325 and a TFZ of 59. Refinement of the structure over 98 rounds was based on 36 368 reflections, with 1814 reflections (5%) set aside for *R*_free_. *PDB-REDO* (Joosten *et al.*, 2014 ▸) was used at an intermediate refinement stage and improved the fit from an *R*_work_ of 0.2293 and an *R*_free_ of 0.2615 to an *R*_work_ of 0.2281 and an *R*_free_ of 0.2590. The final structure had an *R*_work_ of 0.2202 and an *R*_free_ of 0.2520. As with HNL40, there were two protein monomers in the asymmetric unit of HNL71. The dimer consists of 497 protein residues, 59 solvent molecules and a total of 3795 non-H atoms. The average *B* factor for the final model was 30.15 Å^2^. Structural validation using *MolProbity* showed 95.86% of the residues in favored regions and 4.14% in the allowed regions of the Ramachandran plot, with no outliers (Table 4 ▸).

As is common in X-ray structures, the N-terminal His_6_-tag and linker in HNL40 and the C-terminal His_6_-tag and linker in HNL71 were not seen in the electron density. In HNL71, the data also lacked sufficient electron density to define nine residues in two surface regions: a two-residue segment Gly142-Lys143 in the cap domain and a seven-residue segment Met182-Glu183-Ile184-Leu185-Ala186-Lys187-Arg188 in the catalytic domain. We hypothesize that these regions are flexible and adopt multiple conformations in the crystal and in solution.

**Figure 3:**
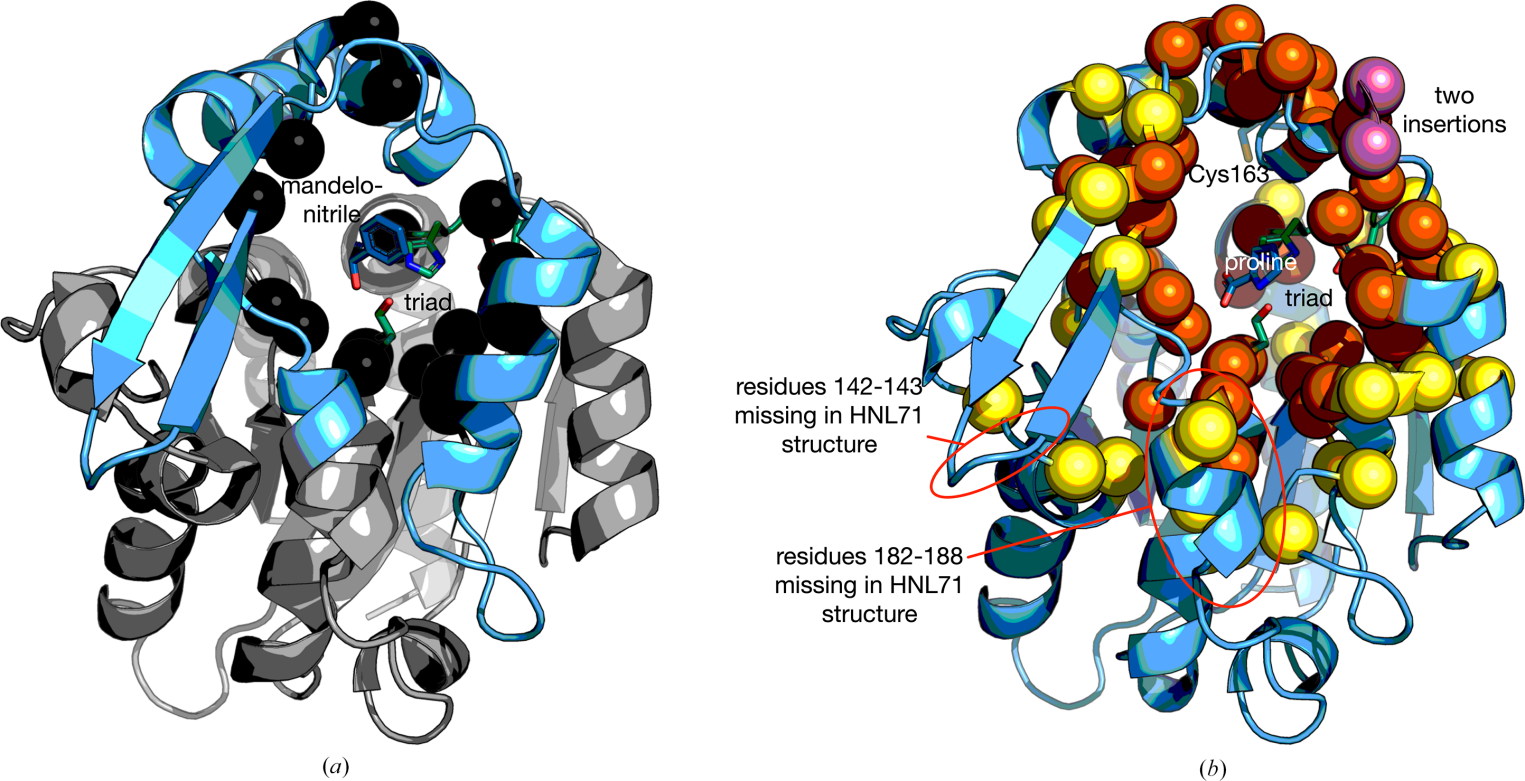
Location of the changes in *Hb*HNL to make increasingly large regions surrounding the active site identical in sequence to the esterase SABP2. (*a*) Ribbon diagram of *Hb*HNL (PDB entry 1yb6) showing the locations of changes introduced into *Hb*HNL to create HNL16. The lid domain (sky blue, 108–181) lies at the top and front, while the catalytic domain (light gray, 1–107 and 182–257) lies in the lower and deeper parts in this view. The catalytic triad Ser–His–Asp (side chains shown as green sticks) lies in the catalytic domain at the interface between the two domains. The *Hb*HNL substrate mandelonitrile (blue sticks) lies in the active site. HNL16 contains 16 substitutions at the locations marked by black spheres. These changes make the amino-acid residues in HNL16 identical to those in SABP2 in a region surrounding the substrate by ∼6.5 Å. (*b*) Ribbon diagram of chain *A* of HNL40 (PDB entry 8sni) showing the locations of changes introduced into *Hb*HNL to create HNL40 and HNL71. The HNL40 set includes the substitutions in HNL16. A bound l-proline (blue sticks) lies in the substrate-binding region and mimics the product carboxylate of an ester hydrolysis. Both HNL40 and HNL71 include two insertions (rose spheres) after residue 126 of *Hb*HNL and the 38 substitutions marked as dark orange spheres. These changes in HNL40 make the protein sequence of the region within 10 Å of the substrate-binding site identical to that in SABP2. HNL71 contains 31 additional substitutions (yellow spheres) to make the protein sequence of the region within 14 Å identical to that in SABP2. In the HNL71 structure, the electron density was insufficient to define the positions of nine surface-exposed amino-acid residues: 142–143 (lid domain) and 182–188 (catalytic domain). These regions were defined in the HNL40 structure shown and are circled in red. In HNL71, the side chain of Cys163 (sticks) is oxidized to the sulfenic acid.

The structures of hydroxynitrile lyase *Hb*HNL and variants HNL40 and HLN71 are similar and consist of two domains (Fig. 3 ▸). According to the *Hb*HNL numbering, the catalytic domain (1–107 and 180–265) contains the catalytic triad (Ser80–Asp207–His237) and adopts the α/β-hydrolase fold, while the lid domain (108–179) forms part of the active-site region. Fig. 3 ▸(*a*) shows the locations of the substitutions that created HNL16 from *Hb*HNL superimposed on the structure of *Hb*HNL. Fig. 3 ▸(*b*) shows the locations of the substitutions and insertions that created HNL40 and HNL71 from *Hb*HNL superimposed on the structure of HNL40. The 38 substitutions in HNL40 surround the active site and the two inserted amino acids lie on the surface in the lid domain. The additional substitutions in HNL71 further extend the region of sequence identity between SABP2 and the variant protein. Many of the changes occur on the protein surface in the lid since many of these residues lie with 10–14 Å of the active site.

### 3.3. Catalytic residue positions

The catalytic residue positions were compared in two ways (Table 6 ▸). The C^α^-atom positions compared the main-chain positions, while the catalytic atom positions compared the side-chain positions for the triad (Ser O^γ^, His N^ɛ2^ and Asp O^δ2^) and the main-chain nitrogen positions for the two oxyanion-hole residues. The differences were similar for both the C^α^ and the catalytic atom comparison, with the exception of Ser O^γ^, which showed more variation than Ser C^α^ in both the SABP2 and *Hb*HNL structures due to side-chain flexibility. Three structures of SABP2 showed an average deviation of 0.3 ± 0.1 Å in the C^α^-atom positions and 0.5 ± 0.6 Å for the catalytic atom positions. Deviations of the atom positions by these amounts or less will be considered to be positions indistinguishable from those in SABP2.

The catalytic residue positions in *Hb*HNL (three ligand-free structures) differ from those in SABP2 by 0.8 ± 0.4 Å for the C^α^-atom positions and 0.8 ± 0.3 Å for the catalytic atom positions (Table 5 ▸ and Fig. 2 ▸*a*). The largest deviation is in the position of C^α^ and N of the OX1 residue: 1.2–1.3 Å. The other large deviation is the χ_1_ angle at OX2, which differs by 124° from that in SABP2. These large differences likely create a nonfunctional oxyanion hole in *Hb*HNL.

For HNL40, the average C^α^-atom positions, but not the catalytic atom positions, match those in SABP2 (Fig. 2 ▸*b*, Table 6 ▸). The average C^α^-atom distance, 0.3 ± 0.2 Å, is similar to that for comparison of the three SABP2 structures with each other. The χ_1_ angle at OX2 is much closer to that in SABP2. However, the average catalytic atom distance of 0.8 ± 0.9 Å is larger than in the comparison of SABP2 structures with each other: 0.5 ± 0.6 Å. The largest deviations are 2.3 Å for Ser O^γ^ and 0.84 Å for His N^ɛ2^. Thus, the 40 changes introduced to create HNL40 appear to have restored the oxyanion-hole and catalytic atom positions to be more similar to those in SABP2, but not identical.

For HNL71, both the average C^α^-atom positions and the catalytic atom positions match those in SABP2 (Fig. 2 ▸*c*, Table 6 ▸). The average distances between HNL71 and SABP2 are smaller than those for a comparison of three SABP2 structures with each other. The χ_1_ angles at histidine, serine and OX2 are within 7° of those in SABP2. However, comparison of the individual distances suggests that the His N^ɛ2^ and OX1 N positions in HNL71 still differ from those in SABP2. Thus, the 71 changes introduced to create HNL71 create nearly, but not completely, identical catalytic atom positions.

### 3.4. l-Proline bound in the active site of HNL40

The structure of HNL40 contains an l-proline bound in the active site (Fig. 3 ▸). Supplementary Fig. S2 shows the electron-density map that defines the bound l-proline. The l-proline originated from the crystallization solution, which contained 0.2 *M*l-proline, and mimics a bound carboxylate product of an esterase reaction. At the near-neutral pH of the crystallization solution, proline is expected to be zwitterionic with a negatively charged carboxylate (p*K*_a_ 1.99) and a positively charged ammonium group (p*K*_a_ 10.96).

**Figure 4:**
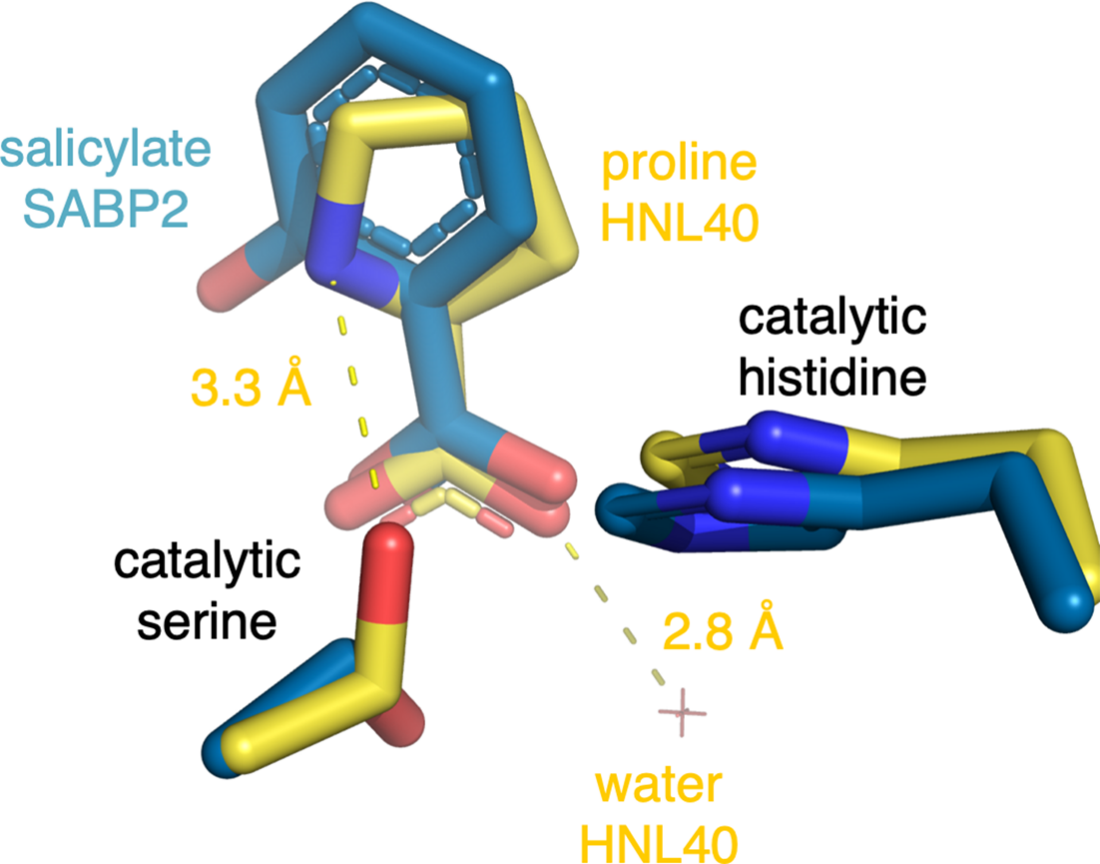
Overlay of the structure of SABP2 with a bound product salicylate (PDB entry 1y7i, blue C atoms) onto the structure of HNL40 with a bound proline (PDB entry 8sni, yellow C atoms). The carboxylate moieties interact with the corresponding protein active sites slightly differently. The catalytic serine donates hydrogen bonds (not shown) to both salicylate carboxylate O atoms (3.0 and 3.0 Å) in the SABP2 structure, but the altered catalytic serine side-chain conformation in the HNL40 structure makes the angle too acute for the serine to donate a hydrogen bond to the proline carboxylate even though the O–O distance is still favorable (3.0 and 3.0 Å). Instead, the one proline carboxylate O atom accepts a hydrogen bond from a nearby water molecule (2.8 Å) and the proline ammonium donates a weak hydrogen bond to the catalytic serine (3.3 Å).

In the SABP2 structure containing a bound salicylate, the salicylate carboxylate accepts hydrogen bonds from the catalytic atoms (Fig. 4 ▸, Supplementary Table S2). The catalytic serine O^γ^ donates hydrogen bonds to both carboxylate oxygens, the catalytic histidine N^ɛ2^ donates a hydrogen bond to one carboxylate oxygen, and the two oxyanion-hole amino hydrogens donate hydrogen bonds to the other carboxylate oxygen.

In the HNL40 structure containing the bound proline, the hydrogen-bond pattern with the catalytic serine differs because the catalytic serine O^γ^ adopts a different conformation. It points towards the ammonium N atom of the proline (3.3 Å) and now lies above the plane defined by the O–C–O of the proline carboxylate. This orientation relative to the lone pairs on the oxygens is too acute for a hydrogen bond. Instead, a water molecule donates a hydrogen bond to one of the carboxylates (2.8 Å). These differences in hydrogen-bonding pattern between SABP2 and HNL40 appear to be due to the ammonium ion in proline rather than due to differences in the active-site structure. Supplementary Table S2 lists the relevant distances between atoms.

### 3.5. Similarity of the 10 Å region surrounding the active site to the corresponding region in SABP2

As expected, the main-chain positions of the *Hb*HNL variants become more similar to those of SABP2 as the sequence identity of the *Hb*HNL variants to SABP2 increases (Table 7 ▸). Firstly, three different SABP2 structures were aligned with each other, which yielded an average r.m.s.d. of 0.30 ± 0.05 Å over 216–235 of the 256 C^α^ atoms. The C^α^ positions of SABP2 and *Hb*HNL differ by more than twice this value: 0.64 Å over 227 out of 253 C^α^ atoms. The differences between SABP2 and HNL40 are smaller at 0.51 Å, and those for HNL71 are even smaller at 0.41 Å. At the same time, the main-chain positions in HNL40 and HNL71 move away from their original positions in *Hb*HNL. The r.m.s.d. between *Hb*HNL and HNL40 is 0.40 Å and increases to 0.47 Å in HNL71. HNL40 and HNL71 differ from each other with an r.m.s.d. of 0.30 Å.

**Figure 5:**
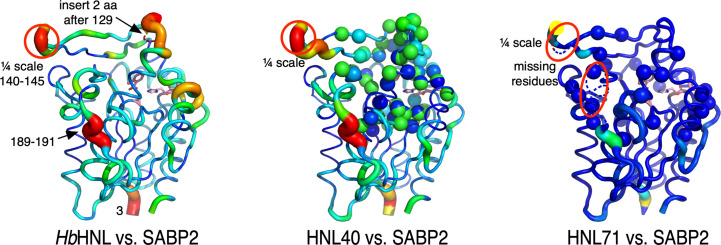
Displacement of C^α^ atoms (ΔC^α^) in *Hb*HNL (PDB entry 1yb6), HNL40 (PDB entry 8sni) and HNL71 (PDB entry 9clr) relative to wild-type SABP2. While the structures of SABP2 and *Hb*HNL differ significantly, the addition of 40 changes to *Hb*HNL to create HNL40 makes the region near the active site more similar to SABP2 and the 71 changes in HNL71 make this region identical to SABP2. All three images show the structure of SABP2 (PDB entry 1y7i), but the color and cartoon thickness indicate the displacement of the C^α^ atoms in the other structures. The triad and bound salicylic acid are shown as pink sticks. The representations are scaled so that a red, thick sausage indicates a displacement of ∼3 Å in all three structures, with the exception of residues 136–141 (circled) in the lid region of SABP2. The C^α^ positions in this region differ by up to 11 Å from those in *Hb*HNL, so this representation was scaled down for clarity. The dotted line near the top in the *Hb*HNL comparison indicates the two-amino-acid insertion in SABP2 that has no counterpart in *Hb*HNL. The two dotted-line regions in the HNL71 comparison (marked by red ovals) were not observed in the X-ray structure, suggesting disorder. The HNL40 comparison shows the C^α^ atoms as spheres at the locations of the 40 changes. The HNL71 comparison shows its additional 31 substitutions as spheres at the C^α^ positions.

The largest differences in C^α^ positions between *Hb*HNL and SABP2 occur in four regions (Fig. 5 ▸). The largest difference, 11.1 Å, occurs within a lid-domain loop at Pro142 in SABP2, which corresponds to Asp139 in *Hb*HNL. This difference and the differences for residues 140–145 in this loop are so large that they are divided by four in order to create the images in Fig. 5 ▸ so that the other differences in the C^α^ positions are also clearly visible. The second region showing large differences between the C^α^ positions occurs near the two-amino-acid insertion (Ala130-Glu131 in SABP2). The C^α^ positions at the amino acid before the insertion, Pro129 in SABP and Pro126 in *Hb*HNL, differ by 5.6 Å. Fig. 5 ▸ shows the inserted amino acids as a dotted line because there is no corresponding amino acid in *Hb*HNL, making ΔC^α^ undefined. The third region showing large differences between the C^α^ positions is surface loop 188–195 in SABP (185–191 in *Hb*HNL). The loop conformation differs, with the largest C^α^ difference (∼3 Å) occurring at Ala189-Lys190-Tyr191 in SABP2. Finally, the C^α^ positions of the N- and C-termini of the two proteins differ by 6 Å at Glu3 of SABP2 and by 3 Å at Asn260 of SABP2.

Three other regions with smaller ΔC^α^ differences between *Hb*HNL and SABP2 are noteworthy because they involve the catalytic residues. The C^α^ positions of oxyanion-hole residue OX1 (Ala13 in SABP2; Ile12 in *Hb*HNL) differ by 1.3 Å. The C^α^ position of residue Asp237 in SABP2 near the catalytic His238 differs by 1.8 Å from the corresponding Asp234 in *Hb*HNL. The C^α^ positions of residues 208–209 next to the catalytic Asp210 differ by 2.0 Å, and residues 215–216, which are adjacent to 208–209, differ by 2.2 and 2.0 Å.

Adding two insertions and 38 substitutions to create HNL40 significantly reduces the differences between it and SABP2 near the two-amino-acid insertion and in all regions involving the catalytic residues (Fig. 5 ▸). The differences at the lid-domain loop at SABP2 Pro142, the surface loop 188–195 in SABP2 and the N- and C-termini remain largely unchanged. Adding an additional 31 substitutions to create HNL71 further reduces differences near the two-amino-acid insertion and all regions involving the catalytic residues (Fig. 5 ▸). The X-ray structure did not resolve part of the lid-domain loop at SABP2 Pro142 and surface loop 188–195 in SABP2, suggesting that these regions are disordered.

To quantify the similarities of the C^α^ positions in the active site and surrounding regions, we aligned the 59 residues that lie within 10 Å of the salicylic acid bound in the active site of SABP2 (Table 8 ▸). The similarity of the main-chain positions between SABP2 and *Hb*HNL in the 10 Å surrounding the active site, 0.62 Å, remains similar to that when comparing the entire proteins, 0.64 Å (Table 6 ▸). The alignment of the C^α^ positions in the 10 Å region surrounding the active site is closer between SABP2 and HNL40 (0.38 Å) and HNL71 (0.28 Å) than it is overall: 0.51 and 0.41 Å, respectively. Conversely, the C^α^ positions between *Hb*HNL and the variants in the 10 Å region are more different: 0.60 Å for HNL40 and 0.56 Å for HNL71 compared with 0.40 and 0.47 Å overall, respectively. A comparison of the changes between *Hb*HNL and HNL40 and HNL71 show that the C^α^ atoms in this region by have moved by an average of ∼0.6 Å. An alignment of three X-ray crystal structures of SABP2 shows an r.m.s.d. of 0.24 ± 0.07 Å in this region, so the positions of the C^α^ atoms in HNL71, but not HNL40, are indistinguishable from different structures of SABP2.

### 3.6. More distant regions

**Figure 6:**
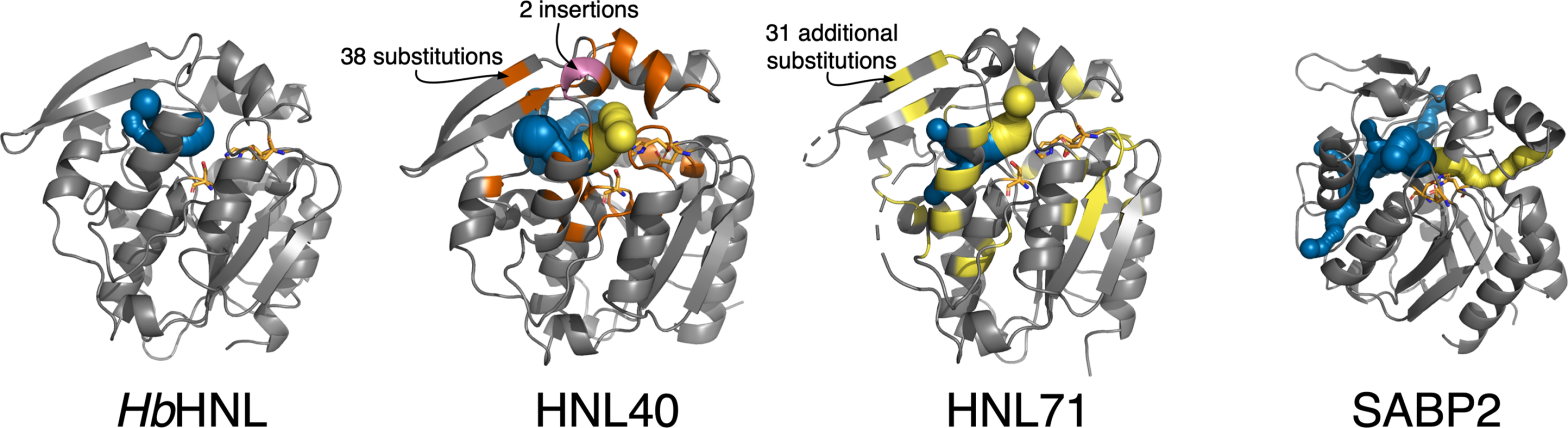
Substitutions in HNL40 and HNL71 create an SABP2-like tunnel (yellow) that is missing in *Hb*HNL. *Hb*HNL (far left) contains two tunnels (blue) that lead from the active site to the left. The catalytic triad is shown as sticks with orange C atoms. In contrast, SABP2 (far right) contains seven tunnels; six of these tunnels (blue) lead from the active site to the left, while one tunnel (yellow) leads from the active site to the right. HNL40 and HNL71 each contain four tunnels; three of these tunnels (blue) lead from the active site to the left, while one tunnel (yellow, near the insertion site) leads from the active site to the right. The 39 substitutions in HNL40 are colored orange, while the two insertions are colored pink. The additional 31 changes in HNL71 are shown in yellow. Tunnels were identified using *Caver Web* (Stourac *et al.*, 2019 ▸) with default settings.

Another structural change introduced by the changes in HNL40 and HNL71 is the creation of additional tunnels from the active site to the protein surface. This change makes the pattern of tunnels in HNL40 and HNL71 intermediate between those in *Hb*HNL and SABP2 (Fig. 6 ▸). *Hb*HNL contains two tunnels, both of which lead to the left in the orientation shown in Fig. 6 ▸. In contrast, SABP2 contains seven tunnels. Six of these tunnels lead to the left and one leads to the right. HNL40 and HNL71 each contain four tunnels, three of which lead to the left and one of which leads to the right.

The new tunnel to the right appears near the insertion site (after residue Pro126) of two amino acids in HNL40. The secondary structure of the main chain in this region changes from a loop to a helix for the two inserted residues (Ala127-Glu128) and the two subsequent residues Asn129-Trp130. SABP2 also contains a helix in this region. While this new tunnel to the right in HNL40 and HNL71 creates a tunnel where none existed in *Hb*HNL, the tunnel differs from the corresponding tunnel in SABP2. In HNL40 and HNL71 the tunnel is short and exits to the front in the view shown in Fig. 6 ▸. In contrast, in SABP2 the tunnel is longer and exits to the back and right.

### 3.7. Sulfenic acid at position 163 in HNL71

**Figure 7:**
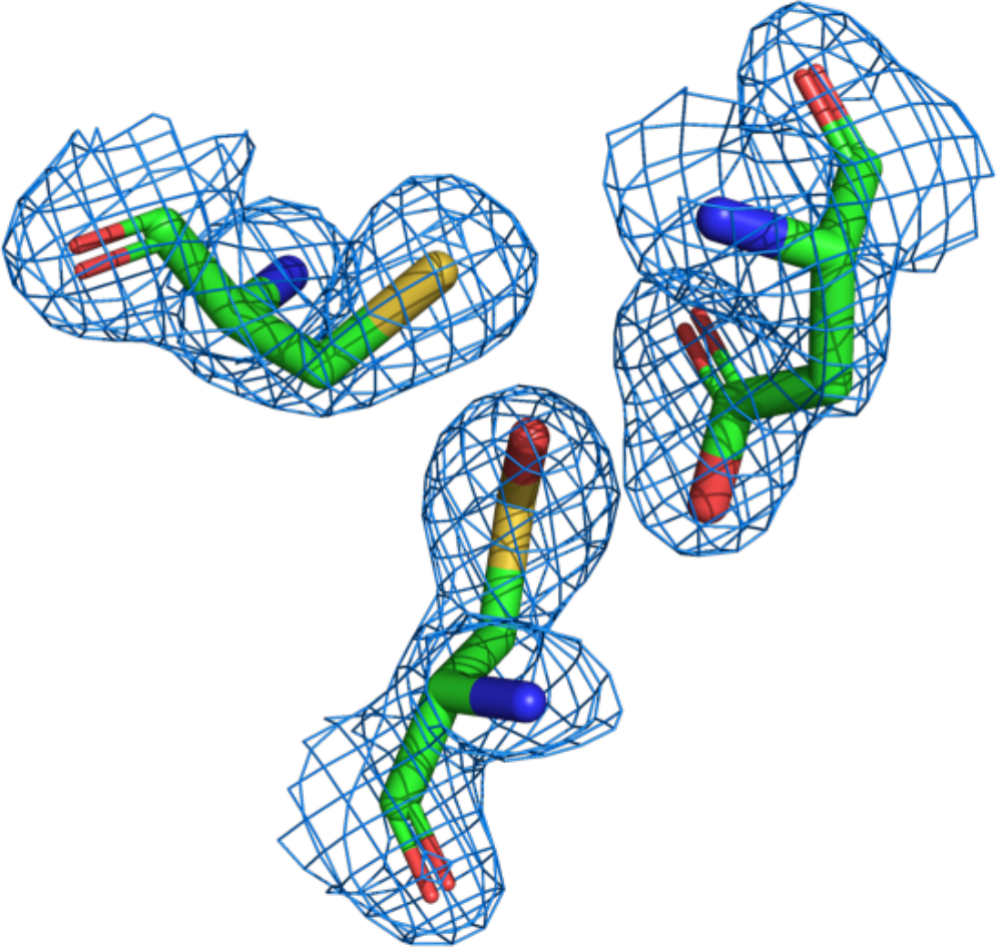
Electron-density map showing the sulfenic acid Cso163 (bottom), Asp236 (top right) and Cys242 (top left) in HNL71. The 2*F*_o_ − * F*_c_ map (blue mesh) is contoured at 1.5σ. No oxidation of the corresponding cysteine was observed in the structures of SABP2, *Hb*HNL or HNL40.

SABP2, *Hb*HNL, HNL71 and HNL40 all contain a cysteine at the position corresponding to residue 164 in SABP2. During refinement of HNL71, the *F*_o_ − *F*_c_ (red/green) electron-density map revealed a notable density adjacent to the sulfur of Cys163 in both chains *A* and *B*. Placing an O atom into this density decreased the *R*_work_ and *R*_free_ values. This addition of an O atom suggests that Cys163 has been oxidized to a sulfenic acid in HNL71 (Fig. 7 ▸). The structures of SABP2, *Hb*HNL and HNL40 show no evidence for oxidation of the corresponding cysteine.

The propensity of a cysteine residue for oxidation is sometimes correlated with the nature of nearby amino acids (Garrido Ruiz *et al.*, 2022 ▸; Salsbury *et al.*, 2008 ▸), but in the case of HNL71 no oxidation-promoting nearby residue could be identified. The closest residue, Asp236, also occurs in SABP2, *Hb*HNL and HNL40. Comparison of the amino acids within 5 Å of the sulfenic acid at position 163 in HNL71 with the corresponding amino acids in SABP2, *Hb*HNL and HNL40, where a similar oxidation was not observed, did not identify an amino acid present in HNL71 that was absent from the other three (Supplementary Table S3). The oxidation seen in HNL71 may be due to oxidative conditions encountered by the HNL71 crystal.

## 4. Conclusions

Despite the similar fold and overall 44% sequence identity between SABP2 and *Hb*HNL, extensive amino-acid changes (40–71 substitutions or insertions) were required to convert *Hb*HNL into an efficient esterase. The 16 substitutions that made the active-site sequences the same did not yield an efficient esterase. Efficient esterase activity required changes in distantly located amino acids, which do not contact the substrate, but must act indirectly. The X-ray crystal structures of HNL40 and HNL71 show two structural changes that may contribute to the increase in esterase activity. Firstly, both HNL40 and HNL71 contain a restored oxyanion hole. The main chain moves the amino nitrogen position of OX1 (Ile12Ala) by 0.9–1.0 Å to better match the position in SABP2, and the side-chain conformation of OX2 (Cys81Leu) changes to allow the substrate to access the oxyanion hole. These structural changes are expected to better stabilize the esterase transition state. Although HNL16 contained both of these substitutions, the additional substitutions added in HNL40 and HNL71 are likely to contribute to the adoption of the new conformations. Secondly, both HNL40 and HNL71 contain a new tunnel similar to one that exists in SABP2 but not in *Hb*HNL. One possible role of this tunnel is to allow the alcohol (*p*-nitrophenol) to exit the active site after formation of the acetyl-enzyme intermediate.

Most of the increase in catalytic turnover occurred from the 24 changes introduced to convert HNL16 to HNL40 (a 210-fold increase in *k*_cat_). These changes occurred between 6.5 and 10 Å from the substrate, which is beyond direct contact with the substrate. If one imagines a layer of amino acids that can contact the substrate, then these 24 changes are in a more distant, second layer that contacts the first layer of amino acids but not the substrate directly. These second-layer amino acids must act indirectly on catalysis. The 31 substitutions to convert HNL40 to HNL71 yielded only a 4.2-fold additional increase, although it is notable that HNL71 is a better esterase (2.4-fold higher *k*_cat_) towards *p*-nitrophenyl acetate than SABP2.

These experiments demonstrated that 40 changes (38 substitutions and two insertions) are sufficient to convert *Hb*HNL into an efficient esterase. Due to the large number of changes it is difficult to assign individual contributions to each change. It is possible, and even likely, that not all of these changes are required, so a smaller subset of changes might also create an efficient esterase. Such a smaller subset would be useful to understand the individual roles of the changes.

## Supplementary Material

PDB reference: hydroxynitrile lyase from *Hevea brasiliensis*, with 40 mutations, 8sni

PDB reference: with 71 mutations, 9clr

Supplementary Tables and Figures. DOI: 10.1107/S2059798325007065/chr5006sup1.pdf

## Figures and Tables

**Table 1:** Protein-production information

	HNL40	HNL71
Source organism	*Hevea brasiliensis*	*Hevea brasiliensis*
DNA source	Synthetic construct	Synthetic construct
Expression vector	pET-21a(+)	pET-21a(+)
Expression host	*Escherichia coli* BL21	*Escherichia coli* BL21
Complete amino-acid sequence of the construct produced	MGSSHHHHHHSSGLVPRGSHMASMTGGQQMGRGSMAFAHFVLVHGACHGAWSWHKLKPLLEALGHKVTALDLAASGVDPRQIEEIGSFDEYSEPLLTFLEALPPGEKVILVGHSLGGMNIAIAADKYCEKIAAAVFLAAFLPDTEHCPSYVLDKYNEVTPAENWLDTTFFTYTKDGKEITGMFFGPKFLAHKLYQLCGPEEYELAKMLVRKSSLFMNILAKRPFFTKEGYGSIKKIYVWTDQDKGIPPEFQLWQIENYKPDKVYKVEGGDHMAMLTKTKEIAEILQEVADTYN	MAFAHFVLVHGACHGGWSWHKLKPLLEALGHKVTALDLAASGVDPRQIEELGTLDEYTEPLLTFLEALPPGEKVILVGHSLGGMNLGIAADKYCEKIAAAVFLAAFMPDTEHCSSFVLEQYNERTPAENWLDTQFLTYTKDGKEITSMFFGPKFLAHKLYQLCGPEDLELASMLVRPSSLMMEILAKRPFFTKEGYGSIKKIYIVCTEDKGIPEEFQRWQIENYKPDKVYKVEGADHMAMLCKTKELAEILQEVADTYNAAALEHHHHHH
Codon-optimized coding DNA sequence (CDS)	ATGGGCAGCAGCCATCATCATCATCATCACAGCAGCGGCCTGGTGCCGCGCGGCAGCCATATGGCTAGCATGACTGGTGGACAGCAAATGGGTCGCGGATCCATGGCCTTTGCGCACTTTGTTCTTGTACACGGGGCTTGTCACGGTGCATGGAGCTGGCACAAACTCAAACCACTTCTCGAAGCTCTGGGCCACAAAGTCACGGCTCTTGACTTGGCCGCCAGCGGGGTAGACCCCCGCCAAATTGAAGAGATCGGATCGTTCGACGAGTACAGCGAACCGCTGCTGACTTTTCTGGAAGCATTGCCGCCAGGGGAGAAGGTGATCCTGGTTGGTCACTCTCTGGGCGGAATGAACATTGCCATCGCTGCCGATAAGTACTGCGAGAAGATAGCCGCAGCTGTCTTCTTAGCGGCCTTCTTACCGGACACAGAACACTGCCCAAGTTACGTGTTAGACAAATACAACGAGGTCACCCCAGCGGAGAACTGGTTAGACACCACCTTCTTTACCTACACCAAGGACGGTAAAGAGATTACAGGTATGTTCTTCGGCCCGAAGTTTCTGGCCCATAAGTTATACCAGTTATGCGGCCCAGAAGAATATGAATTGGCGAAGATGCTTGTGCGTAAGTCATCATTATTCATGAACATTTTAGCAAAGCGGCCGTTCTTCACAAAGGAAGGTTACGGCAGTATAAAGAAAATCTATGTGTGGACAGATCAGGACAAGGGGATCCCGCCTGAGTTCCAGCTGTGGCAAATAGAGAATTACAAACCAGACAAGGTTTACAAGGTGGAAGGCGGTGATCACATGGCAATGCTCACTAAGACGAAAGAGATAGCCGAAATCTTGCAAGAGGTCGCTGATACTTATAACTAA	ATGGCATTCGCTCACTTCGTTTTGGTGCATGGTGCATGTCACGGTGGATGGAGTTGGCACAAACTTAAACCCCTTTTGGAAGCTCTCGGTCATAAGGTGACCGCGTTGGACCTGGCGGCAAGCGGTGTAGACCCACGGCAAATCGAGGAACTCGGCACGCTCGACGAGTACACGGAGCCGTTATTGACGTTTCTTGAGGCATTGCCGCCCGGAGAGAAGGTCATACTGGTGGGACACTCACTCGGTGGAATGAATCTTGGTATCGCCGCTGACAAGTATTGCGAAAAGATTGCGGCGGCTGTATTCCTGGCTGCCTTTATGCCGGATACTGAGCACTGTAGCAGCTTCGTATTGGAACAGTACAATGAGCGGACGCCGGCGGAGAATTGGCTGGACACTCAATTTCTGACCTATACTAAGGACGGCAAGGAGATTACTAGTATGTTCTTCGGCCCAAAGTTTCTGGCTCACAAATTATACCAGTTATGCGGTCCTGAGGACCTTGAGCTTGCCAGCATGTTGGTTCGTCCTAGCTCACTGTTCATGGAAATCTTGGCAAAGCGCCCTTTCTTCACCAAGGAAGGCTATGGTAGCATTAAGAAGATCTATATCGTTTGCACGGAAGACAAGGGTATCCCGGAAGAGTTCCAACGTTGGCAGATTGAGAACTACAAACCTGATAAAGTTTATAAAGTAGAGGGTGCAGACCATATGGCGATGCTCTGCAAGACAAAGGAACTCGCCGAGATTCTCCAGGAAGTCGCGGATACCTACAACGCTGCCGCACTCGAGCATCACCACCATCATCACTAA

**Table 2:** Crystallization information

	HNL40	HNL71
Method	Sitting drop	Sitting drop
Plate type	CrystalMation Intelli-Plate low-profile 96-well, Hampton Research	CrystalMation Intelli-Plate low-profile 96-well, Hampton Research
Temperature (K)	298	298
Protein concentration (mg ml^−1^)	8	12
Buffer composition of protein solution	5 m*M* BES buffer pH 7.2	5 m*M* BES buffer pH 7.2
Composition of reservoir solution	0.1 *M* 4-(2-hydroxyethyl)-1-piperazineethanesulfonic acid (HEPES) pH 7.5, 0.2 *M*L-proline, 10%(*w*/*v*) polyethylene glycol 3350	0.1 *M* Tris pH 8.5, 0.2 *M* magnesium chloride hexahydrate, 25%(*w*/*v*) polyethylene glycol 3350
Volume and ratio of drop (µl)	0.2, 1:1	0.2, 1:1
Volume of reservoir (µl)	50	50

**Table 3:** Data collection and processing Values in parentheses are for the highest resolution shell.

	HNL40	HNL71
X-ray source	24-ID-C, APS	BL12-2, SSRL
Wavelength (Å)	0.979	0.979
Temperature (K)	100	100
Detector	EIGER2 S 16M	EIGER 16M PAD
Exposure time (s)	0.2	0.5
Crystal-to-detector distance (mm)	180	270
Angle increment (°)	0.2	0.5
Resolution range (Å)	60.85–1.99 (2.061–1.99)	42.49–1.99 (2.061–1.99)
Space group	*P*2_1_2_1_2_1_	*P*2_1_2_1_2_1_
*a*, *b*, *c* (Å)	76.53, 81.88, 90.93	49.97, 80.75, 135.46
α, β, γ (°)	90, 90, 90	90, 90, 90
Total No. of atoms	4098	3794
Total reflections	144888 (14934)	217465 (10734)
Unique reflections	39159 (3930)	36391 (3148)
Multiplicity	3.70 (3.80)	5.98 (3.41)
Mosaicity (°)	0.2	0.2
Completeness (%)	98.04 (99.17)	94.64 (83.36)
(*I*/σ(*I*))	10.5 (1.3)	7.6 (2.8)
Wilson *B* factor (Å^2^)	36.55	26.88
*R*_merge_	0.066 (0.967)	0.095 (0.877)
*R*_meas_	0.086 (1.244)	0.183 (0.943)
*R*_p.i.m._	0.054 (0.774)	0.066 (0.342)
CC_1/2_	0.997 (0.698)	0.995 (0.804)

**Table 4:** Structure-refinement information for HNL40 and HNL71 Values in parentheses are for the highest resolution shell.

	HNL40	HNL71
Reflections used in refinement	39116 (3923)	36368 (3147)
Reflections used for *R*_free_	2007 (197)	1814 (163)
*R*_work_	0.1987 (0.3155)	0.2202 (0.3248)
*R*_free_	0.2251 (0.3478)	0.2520 (0.3262)
No. of non-H atoms		
Total	4106	3795
Macromolecules	4003	3722
Ligands	35	14
Solvent	68	59
No. of protein residues	517	497
R.m.s.d., bond lengths (Å)	0.008	0.007
R.m.s.d., angles (°)	0.94	0.89
Ramachandran favored (%)	97.27	95.86
Ramachandran allowed (%)	2.73	4.14
Ramachandran outliers (%)	0.00	0.00
Rotamer outliers (%)	0.25	0.56
Clashscore	2.55	3.47
Average *B* factor (Å^2^)		
Overall	39.18	30.15
Macromolecules	39.16	30.13
Ligands	46.01	36.63
Solvent	37.18	29.69

**Table 5:** Steady-state kinetics for hydrolysis of *p*-nitrophenyl acetate by selected enzymes

Variant	Region identical to SABP2†	*k*_cat_ (min^–1^)	*K*_m_ (m*M*)	*k*_cat_/*K*_m_ (min^–1^ *M*^–1^)
SABP2	na	130 ± 4	2.2 ± 0.2	59000
*Hb*HNL	na	0.25 ± 0.02	3.0 ± 0.4	83
HNL3‡	na	1.6 ± 0.1	0.16 ± 0.02	10000
HNL16	6.5 Å	0.35 ± 0.01	0.6 ± 0.2	640
HNL40	10 Å	74 ± 3	7 ± 1	11000
HNL71	14 Å	310 ± 10	2.1 ± 0.1	150000

† Minimum region surrounding the active site that is identical in sequence to SABP2. A bound product salicylic acid defines the active site of SABP2. Residues with at least one atom within the region indicated are identical in the HNL variants; na, not applicable.

‡ Data from Nedrud *et al.* (2014 ▸).

**Table 6:** Deviation of C^α^-atom and catalytic atom positions of the oxyanion-hole and catalytic triad residues between three structures of SABP2 and between SABP2 and *Hb*HNL, HNL40 and HNL71 The SABP2 column compares three X-ray structures of SABP2 with each other (PDB entries 1xkl, 1y7h and 1y7i). The *Hb*HNL column compares three ligand-free structure of *Hb*HNL (PDB entries 6yas, 3c6x and 2g4l) with the SABP2 structure with the smallest bound ligand, thiocyanate (PDB entry 1y7h). The HNL40 column compares HNL40 with the SABP2 structure with bound salicylate (PDB entry 1y7i). The HNL71 column compares HNL71 with the SABP2 structure with the smallest bound ligand, thiocyanate (PDB entry 1y7h).

	Distance (Å )			
	SABP2	*Hb*HNL	HNL40 (PDB entry 8sni)	HNL71 (PDB entry 9clr)
Ser C^α^	0.44 ± 0.09	0.37 ± 0.07	0.18	0.18
His C^α^	0.17 ± 0.05	0.72 ± 0.04	0.57	0.11
Asp C^α^	0.19 ± 0.10	0.84 ± 0.06	0.39	0.22
OX1 C^α^	0.24 ± 0.05	1.34 ± 0.04	0.29	0.42
OX2 C^α^	0.22 ± 0.07	0.58 ± 0.04	0.21	0.30
Average C^α^ distance	0.3 ± 0.1	0.8 ± 0.4	0.3 ± 0.2	0.2 ± 0.1
Ser O^γ^	1.60 ± 0.60	0.80 ± 0.07	2.30	0.12
His N^ɛ2^	0.30 ± 0.10	0.90 ± 0.10	0.84	0.67
Asp O^δ2^	0.25 ± 0.07	0.82 ± 0.09	0.40	0.22
OX1 N	0.25 ± 0.03	1.21 ± 0.02	0.24	0.34
OX2 N	0.33 ± 0.06	0.41 ± 0.02	0.21	0.24
Average catalytic atom distance	0.5 ± 0.6	0.8 ± 0.3	0.8 ± 0.9	0.3 ± 0.2

**Table 7:** R.m.s.d. values for the alignment of all 256 C^α^ atoms in chain *A* of SABP2, *Hb*HNL, HNL40 and HNL71

	SABP2 (PDB entry 1y7i)	*Hb*HNL (PDB entry 1yb6)	HNL40 (PDB entry 8sni)	HNL71 (PDB entry 9clr)
SABP2 (PDB entries 1y7h and 1xkl)	0.30 ± 0.05 Å for 216–235 of 256 C^α^ atoms†	0.64 Å for 227 of 253 C^α^ atoms	0.51 Å for 235 of 255 C^α^ atoms	0.41 Å for 219 of 246 C^α^ atoms
*Hb*HNL		—	0.40 Å for 214 of 256 C^α^ atoms	0.47 Å for 212 of 247 C^α^ atoms
HNL40			—	0.30 Å for 207 of 249 C^α^ atoms

† Three alignments of X-ray crystal structures of SABP2 (PDB entries 1y7i, 1y7h and 1xkl) with one another.

**Table 8:** R.m.s.d. values for the alignment of C^α^ atoms of the 59 residues within 10 Å of salicylic acid in SABP2

	SABP2 (PDB entry 1y7i)	*Hb*HNL (PDB entry 1yb6)	HNL40 (PDB entry 8sni)	HNL71 (PDB entry 9clr)
SABP2	0.24 ± 0.07 Å for 51–58 of 59 C^α^ atoms†	0.62 Å for 50 of 56 C^α^ atoms	0.38 Å for 58 of 59 C^α^ atoms	0.28 Å for 53 of 57 C^α^ atoms
*Hb*HNL		—	0.60 Å for 52 of 56 C^α^ atoms	0.56 Å for 47 of 54 C^α^ atoms
HNL40			—	0.24 Å for 53 of 57 C^α^ atoms

† Alignment of three X-ray crystal structures of SABP2 (PDB entries 1y7i, 1y7h and 1xkl).

## References

[bb1] Emsley, P., Lohkamp, B., Scott, W. G. & Cowtan, K. (2010). *Acta Cryst.* D**66**, 486–501. 10.1107/S0907444910007493 PMC2852313 20383002

[bb2] Forouhar, F., Yang, Y., Kumar, D., Chen, Y., Fridman, E., Park, S. W., Chiang, Y., Acton, T. B., Montelione, G. T., Pichersky, E., Klessig, D. F. & Tong, L. (2005). *Proc. Natl Acad. Sci. USA*, **102**, 1773–1778. 10.1073/pnas.0409227102 PMC547883 15668381

[bb3] Garrido Ruiz, D., Sandoval-Perez, A., Rangarajan, A. V., Gunderson, E. L. & Jacobson, M. P. (2022). *Biochemistry*, **61**, 2165–2176. 10.1021/acs.biochem.2c00349 PMC9583617 36161872

[bb4] Huitema, C. & Horsman, G. (2018). *bioRxiv*, 316588.

[bb5] Joosten, R. P., Long, F., Murshudov, G. N. & Perrakis, A. (2014). *IUCrJ*, **1**, 213–220. 10.1107/S2052252514009324 PMC4107921 25075342

[bb6] Jumper, J., Evans, R., Pritzel, A., Green, T., Figurnov, M., Ronneberger, O., Tunyasuvunakool, K., Bates, R., Žídek, A., Potapenko, A., Bridgland, A., Meyer, C., Kohl, S. A. A., Ballard, A. J., Cowie, A., Romera-Paredes, B., Nikolov, S., Jain, R., Adler, J., Back, T., Petersen, S., Reiman, D., Clancy, E., Zielinski, M., Steinegger, M., Pacholska, M., Berghammer, T., Bodenstein, S., Silver, D., Vinyals, O., Senior, A. W., Kavukcuoglu, K., Kohli, P. & Hassabis, D. (2021). *Nature*, **596**, 583–589. 10.1038/s41586-021-03819-2 PMC8371605 34265844

[bb7] Liebschner, D., Afonine, P. V., Baker, M. L., Bunkóczi, G., Chen, V. B., Croll, T. I., Hintze, B., Hung, L.-W., Jain, S., McCoy, A. J., Moriarty, N. W., Oeffner, R. D., Poon, B. K., Prisant, M. G., Read, R. J., Richardson, J. S., Richardson, D. C., Sammito, M. D., Sobolev, O. V., Stockwell, D. H., Terwilliger, T. C., Urzhumtsev, A. G., Videau, L. L., Williams, C. J. & Adams, P. D. (2019). *Acta Cryst.* D**75**, 861–877. 10.1107/S2059798319011471 PMC6778852 31588918

[bb8] McCoy, A. J., Grosse-Kunstleve, R. W., Adams, P. D., Winn, M. D., Storoni, L. C. & Read, R. J. (2007). *J. Appl. Cryst.* **40**, 658–674. 10.1107/S0021889807021206 PMC2483472 19461840

[bb9] Nedrud, D. M., Lin, H., Lopez, G., Padhi, S. K., Legatt, G. A. & Kazlauskas, R. J. (2014). *Chem. Sci.* **5**, 4265–4277. 10.1039/c4sc01544d PMC4206950 25346843

[bb10] Otwinowski, Z. & Minor, W. (1997). *Methods Enzymol.* **276**, 307–326. 10.1016/S0076-6879(97)76066-X 27754618

[bb99] Pierce, C. T., Greenberg. L. R., Walsh, M. E., Shi, K., Magee, D. J., Aihara, H., Gordon, W., Evans, R. L. & Kazlauskas, R. J. (2025). *Acta Cryst.* F**81**, 398–405. 10.1107/S2053230X25007034 PMC12400193 40864147

[bb11] Salsbury, F. R., Knutson, S. T., Poole, L. B. & Fetrow, J. S. (2008). *Protein Sci.* **17**, 299–312. 10.1110/ps.073096508 PMC2222711 18227433

[bb12] Sievers, F., Wilm, A., Dineen, D. G., Gibson, T. J., Karplus, K., Li, W., Lopez, R., McWilliam, H., Remmert, M., Söding, J., Thompson, J. D. & Higgins, D. G. (2011). *Mol. Syst. Biol.* **7**, 539. 10.1038/msb.2011.75 PMC3261699 21988835

[bb13] Stourac, J., Vavra, O., Kokkonen, P., Filipovic, J., Pinto, G., Brezovsky, J., Damborsky, J. & Bednar, D. (2019). *Nucleic Acids Res.* **47**, W414–W422. 10.1093/nar/gkz378 PMC6602463 31114897

[bb14] Wagner, U. G., Hasslacher, M., Griengl, H., Schwab, H. & Kratky, C. (1996). *Structure*, **4**, 811–822. 10.1016/s0969-2126(96)00088-3 8805565

