# Supplementary material for: Crystal structures of 40- and 71-substitution variants of hydroxynitrile lyase from rubber tree
Source: Acta Crystallogr D Struct Biol. 2025 Aug 27;81(Pt 9):511–23. doi: 10.1107/S2059798325007065 (PMC12400190; doi:10.1107/S2059798325007065)
Supplement: Supplementary file 1 [file d-81-00511-sup1.pdf]

Acta Cryst D
STRUCTURAL BIOLOGY

Volume 81 (2025)

Supporting information for article:

Crystal structures of 40- and 71-substitution variants of hydroxynitrile lyase from rubber tree

Colin T. Pierce, Panhavuth Tan, Lauren R. Greenberg, Meghan E. Walsh, Ke Shi, Alana H. Nguyen, Elyssa L. Meixner, Sharad Sarak, Hideki Aihara, Robert L. Evans and Romas J. Kazlauskas

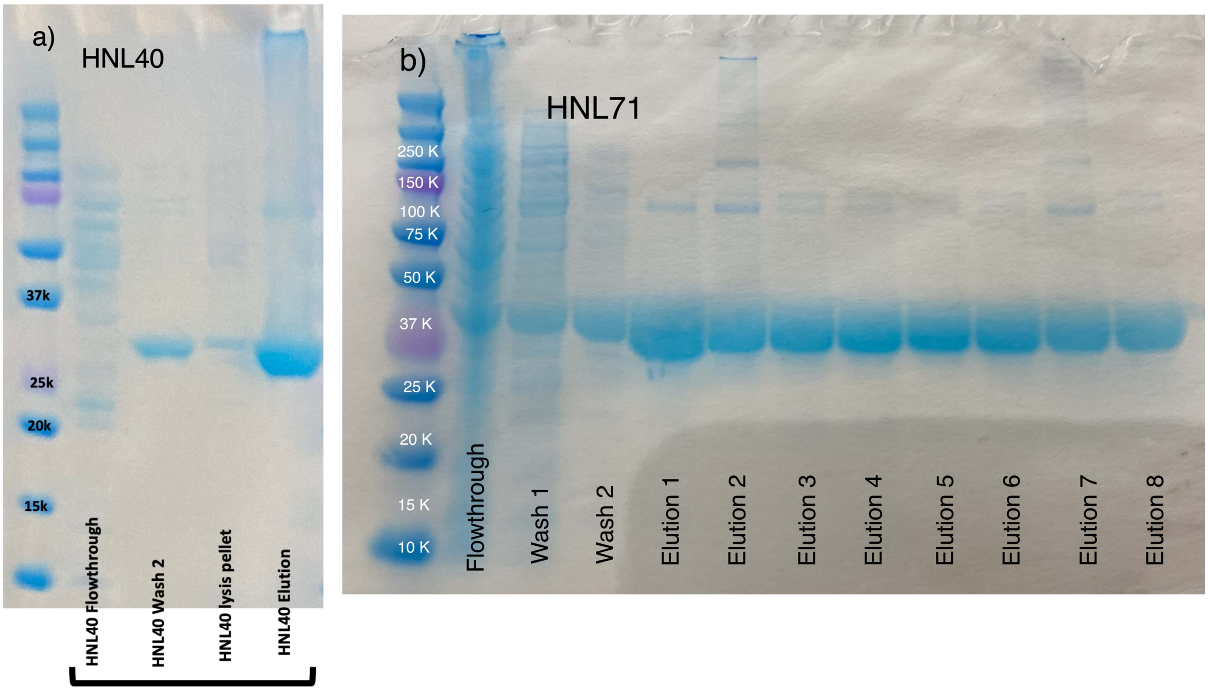

Figure S1 SDS-PAGE showing the purification of a) HNL40 and b) HNL71.

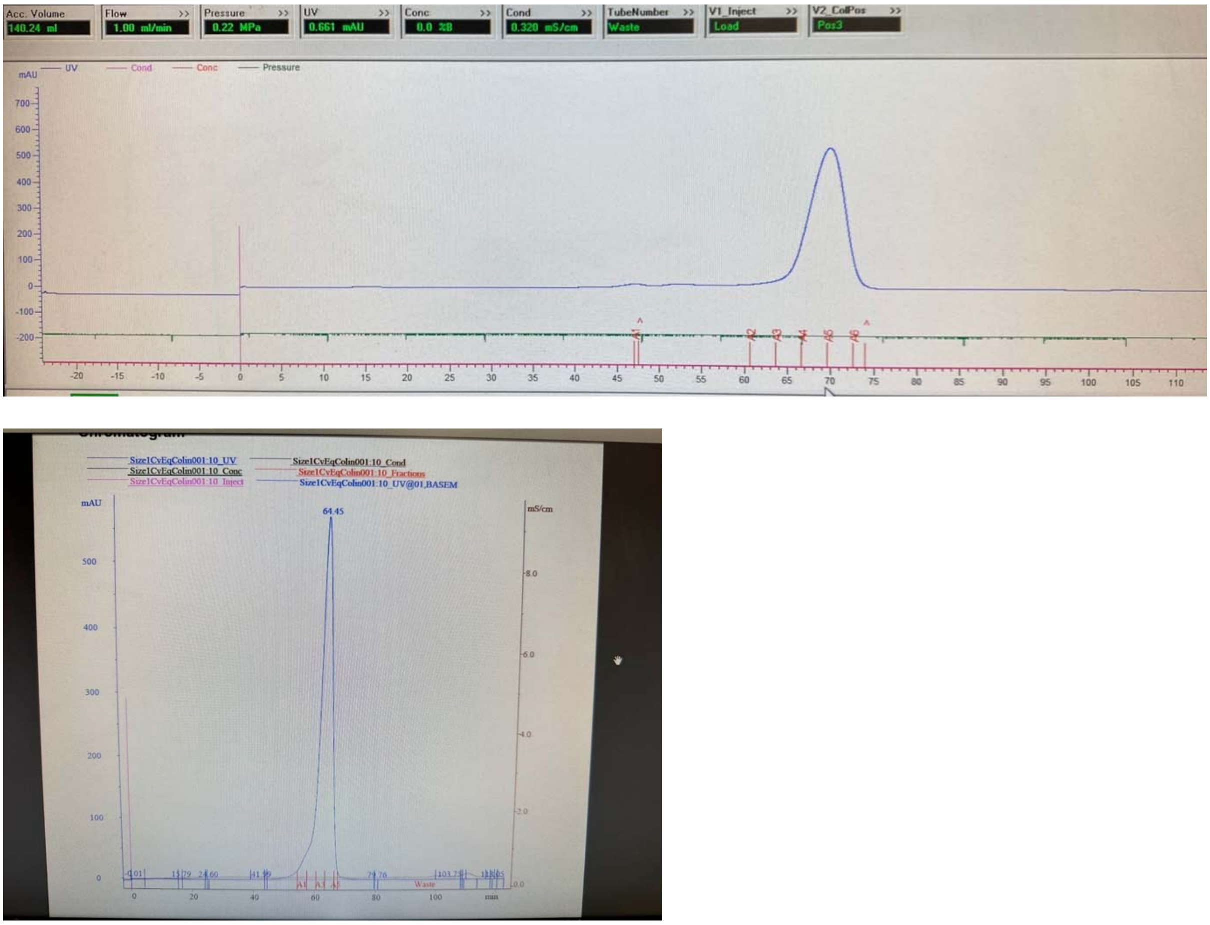

Figure S2 Size-exclusion chromatography traces of HNL71 purification from February 8, 2023 (top) and HNL40 purification from March 19, 2023 (bottom) indicate high purity. UV absorbance at 280 nm (blue trace), given in milli-absorbance units (mAU), indicates the presence of protein eluting as single, symmetrical peaks at ~70 minutes (HNL71) and ~64 minutes (HNL40), consistent with

previously observed elution times for *Hb*HNL-based variants. The absence of additional peaks suggests minimal aggregation or degradation of the protein samples.

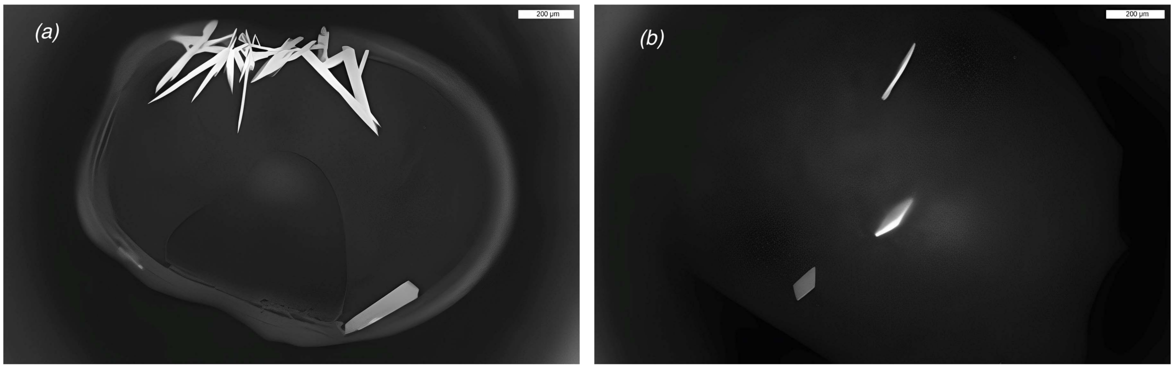

Figure S3 UV fluorescence images of a) HNL40 and b) HNL71 protein crystals taken with a JANSi UVEX imager. Images were captured periodically after plate setup to monitor crystal growth. The intrinsic fluorescence observed under UV illumination originates from five tryptophan residues in HNL40 and four in HNL71. Images were sharpened using post-acquisition image processing to enhance crystal visibility. The protein crystals were barely visible in visible light images.

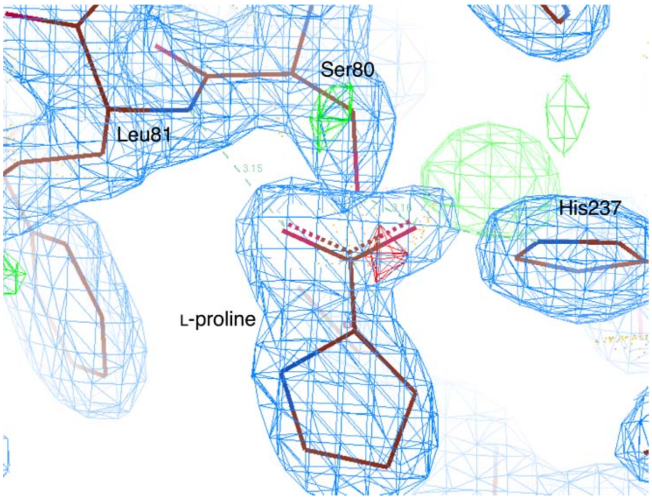

Figure S4 The active site of the HNL40 structure contains a bound L-proline which originates from the crystallization solution and mimics a bound product carboxylate of ester hydrolysis. The electron density map ($2F_O - F_C$ (blue mesh) contoured at 1.5σ) and stick representation of the bound L-proline shows some of the hydrogen bonds that the carboxylate oxygens of the proline accept from the catalytic residues. One carboxylate oxygen of the bound proline accepts hydrogen bonds from the oxyanion hole N-H (Ala12 $d_{N\text{-}O}$ = 2.76 Å (not shown), Leu81 $d_{N\text{-}O}$ = 3.15 Å), while the other carboxylate oxygen accepts hydrogen bonds from the Oɣ-H of the catalytic serine 80 ($d_{O\text{-}O}$ = 3.16 Å), Nε-H of the catalytic histidine 237 ($d_{O\text{-}N}$ = 2.87 Å) and a water molecule 416 ($d_{O\text{-}O}$ = 2.68 Å; not shown). The figure was generated using Coot.

Table S1 DNA sequence of the protein expression plasmids used to make HNL40 and HNL71.

Enzyme	HNL40	HNL71
Source Organism	*Hevea brasiliensis*	*Hevea brasiliensis*
DNA source	synthetic construct	synthetic construct

pET21a (+) backbone sequence	AAGCTTGCGGCCGCACTCGA	GCGGCCGCACTCGAGCACCA
	GCACCACCACCACCACCACT	CCACCACCACCACTGAGATC
	GAGATCCGGCTGCTAACAAA	CGGCTGCTAACAAAGCCCGA
	GCCCGAAAGGAAGCTGAGTT	AAGGAAGCTGAGTTGGCTGC
	GGCTGCTGCCACCGCTGAGC	TGCCACCGCTGAGCAATAAC
	AATAACTAGCATAACCCCTT	TAGCATAACCCCTTGGGGCC
	GGGGCCTCTAAACGGGTCTT	TCTAAACGGGTCTTGAGGGG
	GAGGGGTTTTTTGCTGAAAG	TTTTTTGCTGAAAGGAGGAA
	GAGGAACTATATCCGGATTG	CTATATCCGGATTGGCGAAT
	GCGAATGGGACGCGCCCTGT	GGGACGCGCCCTGTAGCGGC
	AGCGGCGCATTAAGCGCGGC	GCATTAAGCGCGGCGGGTGT
	GGGTGTGGTGGTTACGCGCA	GGTGGTTACGCGCAGCGTGA
	GCGTGACCGCTACACTTGCC	CCGCTACACTTGCCAGCGCC
	AGCGCCCTAGCGCCCGCTCC	CTAGCGCCCGCTCCTTTCGCT
	TTTCGCTTTCTTCCCTTCCTT	TTCTTCCCTTCCTTTCTCGCC
	TCTCGCCACGTTCGCCGGCT	ACGTTCGCCGGCTTTCCCCG
	TTCCCCGTCAAGCTCTAAAT	TCAAGCTCTAAATCGGGGGC
	CGGGGGCTCCCTTTAGGGTT	TCCCTTTAGGGTTCCGATTTA
	CCGATTTAGTGCTTTACGGC	GTGCTTTACGGCACCTCGAC
	ACCTCGACCCCAAAAAACTT	CCCAAAAAACTTGATTAGGG
	GATTAGGGTGATGGTTCACG	TGATGGTTCACGTAGTGGGC
	TAGTGGGCCATCGCCCTGAT	CATCGCCCTGATAGACGGTT
	AGACGGTTTTTCGCCCTTTG	TTTCGCCCTTTGACGTTGGA
	ACGTTGGAGTCCACGTTCTT	GTCCACGTTCTTTAATAGTG
	TAATAGTGGACTCTTGTTCC	GACTCTTGTTCCAAACTGGA
	AAACTGGAACAACACTCAAC	ACAACACTCAACCCTATCTC
	CCTATCTCGGTCTATTCTTTT	GGTCTATTCTTTTGATTTATA
	GATTTATAAGGGATTTTGCC	AGGGATTTTGCCGATTTCGG
	GATTTCGGCCTATTGGTTAA	CCTATTGGTTAAAAAATGAG
	AAAATGAGCTGATTTAACAA	CTGATTTAACAAAAATTTAA
	AAATTTAACGCGAATTTTAA	CGCGAATTTTAACAAAATAT
	CAAAATATTAACGCTTACAA	TAACGCTTACAATTTAGGTG
	TTTAGGTGGCACTTTTCGGG	GCACTTTTCGGGGAAATGTG
	GAAATGTGCGCGGAACCCCT	CGCGGAACCCCTATTTGTTT
	ATTTGTTTATTTTTCTAAATA	ATTTTTCTAAATACATTCAA
	CATTCAAATATGTATCCGCT	ATATGTATCCGCTCATGAGA
	CATGAATTAATTCTTAGAAA	CAATAACCCTGATAAATGCT
	AACTCATCGAGCATCAAATG	TCAATAATATTGAAAAAGGA
	AAACTGCAATTTATTCATAT	AGAGTATGAGTATTCAACAT
	CAGGATTATCAATACCATAT	TTCCGTGTCGCCCTTATTCCC
	TTTTGAAAAAGCCGTTTCTG	TTTTTTGCGGCATTTTGCCTT
	TAATGAAGGAGAAAACTCAC	CCTGTTTTTGCTCACCCAGA

	CGAGGCAGTTCCATAGGATG	AACGCTGGTGAAAGTAAAA
	GCAAGATCCTGGTATCGGTC	GATGCTGAAGATCAGTTGGG
	TGCGATTCCGACTCGTCCAA	TGCACGAGTGGGTTACATCG
	CATCAATACAACCTATTAAT	AACTGGATCTCAACAGCGGT
	TTCCCCTCGTCAAAAATAAG	AAGATCCTTGAGAGTTTTCG
	GTTATCAAGTGAGAAATCAC	CCCCGAAGAACGTTTTCCAA
	CATGAGTGACGACTGAATCC	TGATGAGCACTTTTAAAGTT
	GGTGAGAATGGCAAAAGTTT	CTGCTATGTGGCGCGGTATT
	ATGCATTTCTTTCCAGACTTG	ATCCCGTATTGACGCCGGGC
	TTCAACAGGCCAGCCATTAC	AAGAGCAACTCGGTCGCCGC
	GCTCGTCATCAAAATCACTC	ATACACTATTCTCAGAATGA
	GCATCAACCAAACCGTTATT	CTTGGTTGAGTACTCACCAG
	CATTCGTGATTGCGCCTGAG	TCACAGAAAAGCATCTTACG
	CGAGACGAAATACGCGATCG	GATGGCATGACAGTAAGAG
	CTGTTAAAAGGACAATTACA	AATTATGCAGTGCTGCCATA
	AACAGGAATCGAATGCAACC	ACCATGAGTGATAACACTGC
	GGCGCAGGAACACTGCCAG	GGCCAACTTACTTCTGACAA
	CGCATCAACAATATTTTCAC	CGATCGGAGGACCGAAGGA
	CTGAATCAGGATATTCTTCT	GCTAACCGCTTTTTTGCACA
	AATACCTGGAATGCTGTTTT	ACATGGGGGATCATGTAACT
	CCCGGGGATCGCAGTGGTGA	CGCCTTGATCGTTGGGAACC
	GTAACCATGCATCATCAGGA	GGAGCTGAATGAAGCCATAC
	GTACGGATAAAATGCTTGAT	CAAACGACGAGCGTGACACC
	GGTCGGAAGAGGCATAAATT	ACGATGCCTGCAGCAATGGC
	CCGTCAGCCAGTTTAGTCTG	AACAACGTTGCGCAAACTAT
	ACCATCTCATCTGTAACATC	TAACTGGCGAACTACTTACT
	ATTGGCAACGCTACCTTTGC	CTAGCTTCCCGGCAACAATT
	CATGTTTCAGAAACAACTCT	AATAGACTGGATGGAGGCG
	GGCGCATCGGGCTTCCCATA	GATAAAGTTGCAGGACCACT
	CAATCGATAGATTGTCGCAC	TCTGCGCTCGGCCCTTCCGG
	CTGATTGCCCGACATTATCG	CTGGCTGGTTTATTGCTGAT
	CGAGCCCATTTATACCCATA	AAATCTGGAGCCGGTGAGCG
	TAAATCAGCATCCATGTTGG	TGGGTCTCGCGGTATCATTG
	AATTTAATCGCGGCCTAGAG	CAGCACTGGGGCCAGATGGT
	CAAGACGTTTCCCGTTGAAT	AAGCCCTCCCGTATCGTAGT
	ATGGCTCATAACACCCCTTG	TATCTACACGACGGGGAGTC
	TATTACTGTTTATGTAAGCA	AGGCAACTATGGATGAACGA
	GACAGTTTTATTGTTCATGA	AATAGACAGATCGCTGAGAT
	CCAAAATCCCTTAACGTGAG	AGGTGCCTCACTGATTAAGC
	TTTTCGTTCCACTGAGCGTCA	ATTGGTAACTGTCAGACCAA
	GACCCCGTAGAAAAGATCAA	GTTTACTCATATATACTTTAG
	AGGATCTTCTTGAGATCCTTT	ATTGATTTAAAACTTCATTTT

	TTTTCTGCGCGTAATCTGCTG	TAATTTAAAAGGATCTAGGT
	CTTGCAAACAAAAAAACCAC	GAAGATCCTTTTTGATAATC
	CGCTACCAGCGGTGGTTTGT	TCATGACCAAAATCCCTTAA
	TTGCCGGATCAAGAGCTACC	CGTGAGTTTTCGTTCCACTG
	AACTCTTTTTCCGAAGGTAA	AGCGTCAGACCCCGTAGAAA
	CTGGCTTCAGCAGAGCGCAG	AGATCAAAGGATCTTCTTGA
	ATACCAAATACTGTCCTTCT	GATCCTTTTTTTCTGCGCGTA
	AGTGTAGCCGTAGTTAGGCC	ATCTGCTGCTTGCAAACAAA
	ACCACTTCAAGAACTCTGTA	AAAACCACCGCTACCAGCGG
	GCACCGCCTACATACCTCGC	TGGTTTGTTTGCCGGATCAA
	TCTGCTAATCCTGTTACCAGT	GAGCTACCAACTCTTTTTCC
	GGCTGCTGCCAGTGGCGATA	GAAGGTAACTGGCTTCAGCA
	AGTCGTGTCTTACCGGGTTG	GAGCGCAGATACCAAATACT
	GACTCAAGACGATAGTTACC	GTCCTTCTAGTGTAGCCGTA
	GGATAAGGCGCAGCGGTCG	GTTAGGCCACCACTTCAAGA
	GGCTGAACGGGGGGTTCGTG	ACTCTGTAGCACCGCCTACA
	CACACAGCCCAGCTTGGAGC	TACCTCGCTCTGCTAATCCTG
	GAACGACCTACACCGAACTG	TTACCAGTGGCTGCTGCCAG
	AGATACCTACAGCGTGAGCT	TGGCGATAAGTCGTGTCTTA
	ATGAGAAAGCGCCACGCTTC	CCGGGTTGGACTCAAGACGA
	CCGAAGGGAGAAAGGCGGA	TAGTTACCGGATAAGGCGCA
	CAGGTATCCGGTAAGCGGCA	GCGGTCGGGCTGAACGGGG
	GGGTCGGAACAGGAGAGCG	GGTTCGTGCACACAGCCCAG
	CACGAGGGAGCTTCCAGGGG	CTTGGAGCGAACGACCTACA
	GAAACGCCTGGTATCTTTAT	CCGAACTGAGATACCTACAG
	AGTCCTGTCGGGTTTCGCCA	CGTGAGCTATGAGAAAGCGC
	CCTCTGACTTGAGCGTCGAT	CACGCTTCCCGAAGGGAGAA
	TTTTGTGATGCTCGTCAGGG	AGGCGGACAGGTATCCGGTA
	GGGCGGAGCCTATGGAAAA	AGCGGCAGGGTCGGAACAG
	ACGCCAGCAACGCGGCCTTT	GAGAGCGCACGAGGGAGCT
	TTACGGTTCCTGGCCTTTTGC	TCCAGGGGGAAACGCCTGGT
	TGGCCTTTTGCTCACATGTTC	ATCTTTATAGTCCTGTCGGGT
	TTTCCTGCGTTATCCCCTGAT	TTCGCCACCTCTGACTTGAG
	TCTGTGGATAACCGTATTAC	CGTCGATTTTTGTGATGCTCG
	CGCCTTTGAGTGAGCTGATA	TCAGGGGGGCGGAGCCTATG
	CCGCTCGCCGCAGCCGAACG	GAAAAACGCCAGCAACGCG
	ACCGAGCGCAGCGAGTCAGT	GCCTTTTTACGGTTCCTGGCC
	GAGCGAGGAAGCGGAAGAG	TTTTGCTGGCCTTTTGCTCAC
	CGCCTGATGCGGTATTTTCTC	ATGTTCTTTCCTGCGTTATCC
	CTTACGCATCTGTGCGGTAT	CCTGATTCTGTGGATAACCG
	TTCACACCGCAATGGTGCAC	TATTACCGCCTTTGAGTGAG
	TCTCAGTACAATCTGCTCTG	CTGATACCGCTCGCCGCAGC

	ATGCCGCATAGTTAAGCCAG	CGAACGACCGAGCGCAGCG
	TATACACTCCGCTATCGCTA	AGTCAGTGAGCGAGGAAGC
	CGTGACTGGGTCATGGCTGC	GGAAGAGCGCCTGATGCGGT
	GCCCCGACACCCGCCAACAC	ATTTTCTCCTTACGCATCTGT
	CCGCTGACGCGCCCTGACGG	GCGGTATTTCACACCGCAAT
	GCTTGTCTGCTCCCGGCATC	GGTGCACTCTCAGTACAATC
	CGCTTACAGACAAGCTGTGA	TGCTCTGATGCCGCATAGTT
	CCGTCTCCGGGAGCTGCATG	AAGCCAGTATACACTCCGCT
	TGTCAGAGGTTTTCACCGTC	ATCGCTACGTGACTGGGTCA
	ATCACCGAAACGCGCGAGG	TGGCTGCGCCCCGACACCCG
	CAGCTGCGGTAAAGCTCATC	CCAACACCCGCTGACGCGCC
	AGCGTGGTCGTGAAGCGATT	CTGACGGGCTTGTCTGCTCC
	CACAGATGTCTGCCTGTTCA	CGGCATCCGCTTACAGACAA
	TCCGCGTCCAGCTCGTTGAG	GCTGTGACCGTCTCCGGGAG
	TTTCTCCAGAAGCGTTAATG	CTGCATGTGTCAGAGGTTTT
	TCTGGCTTCTGATAAAGCGG	CACCGTCATCACCGAAACGC
	GCCATGTTAAGGGCGGTTTT	GCGAGGCAGCTGCGGTAAA
	TTCCTGTTTGGTCACTGATGC	GCTCATCAGCGTGGTCGTGA
	CTCCGTGTAAGGGGGATTTC	AGCGATTCACAGATGTCTGC
	TGTTCATGGGGGTAATGATA	CTGTTCATCCGCGTCCAGCT
	CCGATGAAACGAGAGAGGA	CGTTGAGTTTCTCCAGAAGC
	TGCTCACGATACGGGTTACT	GTTAATGTCTGGCTTCTGAT
	GATGATGAACATGCCCGGTT	AAAGCGGGCCATGTTAAGGG
	ACTGGAACGTTGTGAGGGTA	CGGTTTTTTCCTGTTTGGTCA
	AACAACTGGCGGTATGGATG	CTGATGCCTCCGTGTAAGGG
	CGGCGGGACCAGAGAAAAA	GGATTTCTGTTCATGGGGGT
	TCACTCAGGGTCAATGCCAG	AATGATACCGATGAAACGAG
	CGCTTCGTTAATACAGATGT	AGAGGATGCTCACGATACGG
	AGGTGTTCCACAGGGTAGCC	GTTACTGATGATGAACATGC
	AGCAGCATCCTGCGATGCAG	CCGGTTACTGGAACGTTGTG
	ATCCGGAACATAATGGTGCA	AGGGTAAACAACTGGCGGTA
	GGGCGCTGACTTCCGCGTTT	TGGATGCGGCGGGACCAGA
	CCAGACTTTACGAAACACGG	GAAAAATCACTCAGGGTCAA
	AAACCGAAGACCATTCATGT	TGCCAGCGCTTCGTTAATAC
	TGTTGCTCAGGTCGCAGACG	AGATGTAGGTGTTCCACAGG
	TTTTGCAGCAGCAGTCGCTT	GTAGCCAGCAGCATCCTGCG
	CACGTTCGCTCGCGTATCGG	ATGCAGATCCGGAACATAAT
	TGATTCATTCTGCTAACCAG	GGTGCAGGGCGCTGACTTCC
	TAAGGCAACCCCGCCAGCCT	GCGTTTCCAGACTTTACGAA
	AGCCGGGTCCTCAACGACAG	ACACGGAAACCGAAGACCA
	GAGCACGATCATGCGCACCC	TTCATGTTGTTGCTCAGGTCG
	GTGGGGCCGCCATGCCGGCG	CAGACGTTTTGCAGCAGCAG

	ATAATGGCCTGCTTCTCGCC	TCGCTTCACGTTCGCTCGCGT
	GAAACGTTTGGTGGCGGGAC	ATCGGTGATTCATTCTGCTA
	CAGTGACGAAGGCTTGAGCG	ACCAGTAAGGCAACCCCGCC
	AGGGCGTGCAAGATTCCGAA	AGCCTAGCCGGGTCCTCAAC
	TACCGCAAGCGACAGGCCG	GACAGGAGCACGATCATGCG
	ATCATCGTCGCGCTCCAGCG	CACCCGTGGGGCCGCCATGC
	AAAGCGGTCCTCGCCGAAAA	CGGCGATAATGGCCTGCTTC
	TGACCCAGAGCGCTGCCGGC	TCGCCGAAACGTTTGGTGGC
	ACCTGTCCTACGAGTTGCAT	GGGACCAGTGACGAAGGCTT
	GATAAAGAAGACAGTCATA	GAGCGAGGGCGTGCAAGATT
	AGTGCGGCGACGATAGTCAT	CCGAATACCGCAAGCGACAG
	GCCCCGCGCCCACCGGAAGG	GCCGATCATCGTCGCGCTCC
	AGCTGACTGGGTTGAAGGCT	AGCGAAAGCGGTCCTCGCCG
	CTCAAGGGCATCGGTCGAGA	AAAATGACCCAGAGCGCTGC
	TCCCGGTGCCTAATGAGTGA	CGGCACCTGTCCTACGAGTT
	GCTAACTTACATTAATTGCG	GCATGATAAAGAAGACAGTC
	TTGCGCTCACTGCCCGCTTTC	ATAAGTGCGGCGACGATAGT
	CAGTCGGGAAACCTGTCGTG	CATGCCCCGCGCCCACCGGA
	CCAGCTGCATTAATGAATCG	AGGAGCTGACTGGGTTGAAG
	GCCAACGCGCGGGGAGAGG	GCTCTCAAGGGCATCGGTCG
	CGGTTTGCGTATTGGGCGCC	AGATCCCGGTGCCTAATGAG
	AGGGTGGTTTTTCTTTTCACC	TGAGCTAACTTACATTAATT
	AGTGAGACGGGCAACAGCT	GCGTTGCGCTCACTGCCCGC
	GATTGCCCTTCACCGCCTGG	TTTCCAGTCGGGAAACCTGT
	CCCTGAGAGAGTTGCAGCAA	CGTGCCAGCTGCATTAATGA
	GCGGTCCACGCTGGTTTGCC	ATCGGCCAACGCGCGGGGA
	CCAGCAGGCGAAAATCCTGT	GAGGCGGTTTGCGTATTGGG
	TTGATGGTGGTTAACGGCGG	CGCCAGGGTGGTTTTTCTTTT
	GATATAACATGAGCTGTCTT	CACCAGTGAGACGGGCAAC
	CGGTATCGTCGTATCCCACT	AGCTGATTGCCCTTCACCGC
	ACCGAGATATCCGCACCAAC	CTGGCCCTGAGAGAGTTGCA
	GCGCAGCCCGGACTCGGTAA	GCAAGCGGTCCACGCTGGTT
	TGGCGCGCATTGCGCCCAGC	TGCCCCAGCAGGCGAAAATC
	GCCATCTGATCGTTGGCAAC	CTGTTTGATGGTGGTTAACG
	CAGCATCGCAGTGGGAACGA	GCGGGATATAACATGAGCTG
	TGCCCTCATTCAGCATTTGC	TCTTCGGTATCGTCGTATCCC
	ATGGTTTGTTGAAAACCGGA	ACTACCGAGATATCCGCACC
	CATGGCACTCCAGTCGCCTT	AACGCGCAGCCCGGACTCGG
	CCCGTTCCGCTATCGGCTGA	TAATGGCGCGCATTGCGCCC
	ATTTGATTGCGAGTGAGATA	AGCGCCATCTGATCGTTGGC
	TTTATGCCAGCCAGCCAGAC	AACCAGCATCGCAGTGGGAA
	GCAGACGCGCCGAGACAGA	CGATGCCCTCATTCAGCATT

	ACTTAATGGGCCCGCTAACA	TGCATGGTTTGTTGAAAACC
	GCGCGATTTGCTGGTGACCC	GGACATGGCACTCCAGTCGC
	AATGCGACCAGATGCTCCAC	CTTCCCGTTCCGCTATCGGCT
	GCCCAGTCGCGTACCGTCTT	GAATTTGATTGCGAGTGAGA
	CATGGGAGAAAATAATACTG	TATTTATGCCAGCCAGCCAG
	TTGATGGGTGTCTGGTCAGA	ACGCAGACGCGCCGAGACA
	GACATCAAGAAATAACGCCG	GAACTTAATGGGCCCGCTAA
	GAACATTAGTGCAGGCAGCT	CAGCGCGATTTGCTGGTGAC
	TCCACAGCAATGGCATCCTG	CCAATGCGACCAGATGCTCC
	GTCATCCAGCGGATAGTTAA	ACGCCCAGTCGCGTACCGTC
	TGATCAGCCCACTGACGCGT	TTCATGGGAGAAAATAATAC
	TGCGCGAGAAGATTGTGCAC	TGTTGATGGGTGTCTGGTCA
	CGCCGCTTTACAGGCTTCGA	GAGACATCAAGAAATAACG
	CGCCGCTTCGTTCTACCATC	CCGGAACATTAGTGCAGGCA
	GACACCACCACGCTGGCACC	GCTTCCACAGCAATGGCATC
	CAGTTGATCGGCGCGAGATT	CTGGTCATCCAGCGGATAGT
	TAATCGCCGCGACAATTTGC	TAATGATCAGCCCACTGACG
	GACGGCGCGTGCAGGGCCA	CGTTGCGCGAGAAGATTGTG
	GACTGGAGGTGGCAACGCCA	CACCGCCGCTTTACAGGCTT
	ATCAGCAACGACTGTTTGCC	CGACGCCGCTTCGTTCTACC
	CGCCAGTTGTTGTGCCACGC	ATCGACACCACCACGCTGGC
	GGTTGGGAATGTAATTCAGC	ACCCAGTTGATCGGCGCGAG
	TCCGCCATCGCCGCTTCCAC	ATTTAATCGCCGCGACAATT
	TTTTTCCCGCGTTTTCGCAGA	TGCGACGGCGCGTGCAGGGC
	AACGTGGCTGGCCTGGTTCA	CAGACTGGAGGTGGCAACGC
	CCACGCGGGAAACGGTCTGA	CAATCAGCAACGACTGTTTG
	TAAGAGACACCGGCATACTC	CCCGCCAGTTGTTGTGCCAC
	TGCGACATCGTATAACGTTA	GCGGTTGGGAATGTAATTCA
	CTGGTTTCACATTCACCACC	GCTCCGCCATCGCCGCTTCC
	CTGAATTGACTCTCTTCCGG	ACTTTTTCCCGCGTTTTCGCA
	GCGCTATCATGCCATACCGC	GAAACGTGGCTGGCCTGGTT
	GAAAGGTTTTGCGCCATTCG	CACCACGCGGGAAACGGTCT
	ATGGTGTCCGGGATCTCGAC	GATAAGAGACACCGGCATAC
	GCTCTCCCTTATGCGACTCCT	TCTGCGACATCGTATAACGT
	GCATTAGGAAGCAGCCCAGT	TACTGGTTTCACATTCACCA
	AGTAGGTTGAGGCCGTTGAG	CCCTGAATTGACTCTCTTCCG
	CACCGCCGCCGCAAGGAATG	GGCGCTATCATGCCATACCG
	GTGCATGCAAGGAGATGGCG	CGAAAGGTTTTGCGCCATTC
	CCCAACAGTCCCCCGGCCAC	GATGGTGTCCGGGATCTCGA
	GGGGCCTGCCACCATACCCA	CGCTCTCCCTTATGCGACTCC
	CGCCGAAACAAGCGCTCATG	TGCATTAGGAAGCAGCCCAG
	AGCCCGAAGTGGCGAGCCC	TAGTAGGTTGAGGCCGTTGA

	GATCTTCCCCATCGGTGATG	GCACCGCCGCCGCAAGGAAT
	TCGGCGATATAGGCGCCAGC	GGTGCATGCAAGGAGATGGC
	AACCGCACCTGTGGCGCCGG	GCCCAACAGTCCCCCGGCCA
	TGATGCCGGCCACGATGCGT	CGGGGCCTGCCACCATACCC
	CCGGCGTAGAGGATCGAGAT	ACGCCGAAACAAGCGCTCAT
	CTCGATCCCGCGAAATTAAT	GAGCCCGAAGTGGCGAGCCC
	ACGACTCACTATAGGGGAAT	GATCTTCCCCATCGGTGATG
	TGTGAGCGGATAACAATTCC	TCGGCGATATAGGCGCCAGC
	CCTCTAGAAATAATTTTGTTT	AACCGCACCTGTGGCGCCGG
	AACTTTAAGAAGGAGATATA	TGATGCCGGCCACGATGCGT
	CC	CCGGCGTAGAGGATCGAGAT
		CTCGATCCCGCGAAATTAAT
		ACGACTCACTATAGGGGAAT
		TGTGAGCGGATAACAATTCC
		CCTCTAGGATCCGAATTCAA
		ATAAGGAGGAATAAACC

Codon-optimized coding DNA	ATGGGCAGCAGCCATCATCA	ATGGCATTCGCTCACTTCGTT
sequence (CDS)	TCATCATCACAGCAGCGGCC	TTGGTGCATGGTGCATGTCA
	TGGTGCCGCGCGGCAGCCAT	CGGTGGATGGAGTTGGCACA
	ATGGCTAGCATGACTGGTGG	AACTTAAACCCCTTTTGGAA
	ACAGCAAATGGGTCGCGGAT	GCTCTCGGTCATAAGGTGAC
	CCATGGCCTTTGCGCACTTT	CGCGTTGGACCTGGCGGCAA
	GTTCTTGTACACGGGGCTTG	GCGGTGTAGACCCACGGCAA
	TCACGGTGCATGGAGCTGGC	ATCGAGGAACTCGGCACGCT
	ACAAACTCAAACCACTTCTC	CGACGAGTACACGGAGCCGT
	GAAGCTCTGGGCCACAAAGT	TATTGACGTTTCTTGAGGCA
	CACGGCTCTTGACTTGGCCG	TTGCCGCCCGGAGAGAAGGT
	CCAGCGGGGTAGACCCCCGC	CATACTGGTGGGACACTCAC
	CAAATTGAAGAGATCGGATC	TCGGTGGAATGAATCTTGGT
	GTTCGACGAGTACAGCGAAC	ATCGCCGCTGACAAGTATTG
	CGCTGCTGACTTTTCTGGAA	CGAAAAGATTGCGGCGGCTG
	GCATTGCCGCCAGGGGAGAA	TATTCCTGGCTGCCTTTATGC
	GGTGATCCTGGTTGGTCACT	CGGATACTGAGCACTGTAGC
	CTCTGGGCGGAATGAACATT	AGCTTCGTATTGGAACAGTA
	GCCATCGCTGCCGATAAGTA	CAATGAGCGGACGCCGGCG
	CTGCGAGAAGATAGCCGCAG	GAGAATTGGCTGGACACTCA
	CTGTCTTCTTAGCGGCCTTCT	ATTTCTGACCTATACTAAGG
	TACCGGACACAGAACACTGC	ACGGCAAGGAGATTACTAGT
	CCAAGTTACGTGTTAGACAA	ATGTTCTTCGGCCCAAAGTT
	ATACAACGAGGTCACCCCAG	TCTGGCTCACAAATTATACC
	CGGAGAACTGGTTAGACACC	AGTTATGCGGTCCTGAGGAC
	ACCTTCTTTACCTACACCAA	CTTGAGCTTGCCAGCATGTT
	GGACGGTAAAGAGATTACA	GGTTCGTCCTAGCTCACTGTT
	GGTATGTTCTTCGGCCCGAA	CATGGAAATCTTGGCAAAGC
	GTTTCTGGCCCATAAGTTAT	GCCCTTTCTTCACCAAGGAA
	ACCAGTTATGCGGCCCAGAA	GGCTATGGTAGCATTAAGAA
	GAATATGAATTGGCGAAGAT	GATCTATATCGTTTGCACGG
	GCTTGTGCGTAAGTCATCAT	AAGACAAGGGTATCCCGGA
	TATTCATGAACATTTTAGCA	AGAGTTCCAACGTTGGCAGA
	AAGCGGCCGTTCTTCACAAA	TTGAGAACTACAAACCTGAT
	GGAAGGTTACGGCAGTATAA	AAAGTTTATAAAGTAGAGGG
	AGAAAATCTATGTGTGGACA	TGCAGACCATATGGCGATGC
	GATCAGGACAAGGGGATCCC	TCTGCAAGACAAAGGAACTC
	GCCTGAGTTCCAGCTGTGGC	GCCGAGATTCTCCAGGAAGT
	AAATAGAGAATTACAAACCA	CGCGGATACCTACAACGCTG
	GACAAGGTTTACAAGGTGGA	CCGCACTCGAGCATCACCAC
	AGGCGGTGATCACATGGCAA	CATCATCACTAA
	TGCTCACTAAGACGAAAGAG	

	ATAGCCGAAATCTTGCAAGA<br>GGTCGCTGATACTTATAACT<br>AA	

Table S2 Distances between the polar atoms in HNL40 (8SNI) and SABP2 (1Y7I) and a bound carboxylate-containing molecule, proline or salicylate, respectively.

**atom**	**distance in HNL40 (proline)**	**distance in SABP2 (salicylate)**
carboxylate O1	3.0 Å to Ser80 Oɣ (acute angle)<br>2.8 Å His237 Nε2<br>2.8 Å to water molecule	3.0 Å to Ser81 Oɣ<br>2.8 Å to His238 Nε2
carboxylate O2	2.8 Å to Ala12 N<br>3.0 Å to Ser80 Oɣ (acute angle)<br>3.0 Å to Leu81 N	2.8 Å to Ala13 N<br>3.0 Å to Ser81 Oɣ<br>3.6 Å to Leu82 N
proline N	3.3 Å to Ser80 Oɣ	none

Table S3 Residues near CSO163 in HNL71 and the corresponding residues in SABP2, HNL40 and *Hb*HNL cannot account for the oxidation in HNL71, but not the other three proteins. Eight amino acid residues lie with at least one atom within 5 Å of Sɣ of residue 163 in HNL71. Four of these residues (Tyr160, Leu162, Asp236, Leu241) are identical to the corresponding residues in SABP2, *Hb*HNL and HNL40. A comparison of the remaining four residues shows that at least one of the non-oxidized proteins contains the same amino acid as the one present in HNL71. This lack of a unique nearby amino acid suggests that the oxidation was due to oxidative conditions encountered by the HNL71 crystal and not due to the enhanced reactivity of Cys163 in HNL71.

HNL71	SABP2	HNL40	*Hb*HNL
Gly164	Ser165	Gly164	Gly162
Asp167	Asp168	Glu167	Glu165
Met238	Met239	Met238	Lys236
Cys242	Cys243	Thr242	Thr240
